# Assessment of Worldwide Acute Kidney Injury Epidemiology in Neonates: Design of a Retrospective Cohort Study

**DOI:** 10.3389/fped.2016.00068

**Published:** 2016-07-19

**Authors:** Jennifer G. Jetton, Ronnie Guillet, David J. Askenazi, Lynn Dill, Judd Jacobs, Alison L. Kent, David T. Selewski, Carolyn L. Abitbol, Fredrick J. Kaskel, Maroun J. Mhanna, Namasivayam Ambalavanan, Jennifer R. Charlton, the Neonatal Kidney Collaborative, Ayse Akcan Arikan

**Affiliations:** ^1^Stead Family Department of Pediatrics, Division of Nephrology, Dialysis and Transplantation, University of Iowa Children’s Hospital, Iowa City, IA, USA; ^2^Department of Pediatrics, Division of Neonatology, University of Rochester Medical Center, Rochester, NY, USA; ^3^Department of Pediatrics, Division of Nephrology, University of Alabama at Birmingham, Birmingham, AL, USA; ^4^Data Management Center, Division of Biostatistics and Epidemiology, Cincinnati Children’s Hospital Medical Center, Cincinnati, OH, USA; ^5^Department of Neonatology, Centenary Hospital for Women and Children, Canberra Hospital, Australian National University Medical School, Canberra, ACT, Australia; ^6^Department of Pediatrics and Communicable Diseases, Division of Nephrology, C.S. Mott Children’s Hospital, University of Michigan, Ann Arbor, MI, USA; ^7^Department of Pediatrics, Division of Nephrology, Holtz Children’s Hospital, University of Miami, Miami, FL, USA; ^8^Department of Pediatrics, Division of Nephrology, Children’s Hospital at Montefiore, Albert Einstein, Bronx, NY, USA; ^9^Department of Pediatrics, Division of Neonatology, MetroHealth Medical Center, Case Western Reserve University, Cleveland, OH, USA; ^10^Department of Pediatrics, Division of Neonatology, University of Alabama at Birmingham, Birmingham, AL, USA; ^11^Department of Pediatrics, Division of Nephrology, University of Virginia, Charlottesville, VA, USA

**Keywords:** AWAKEN, NKC, neonate, database, KDIGO, acute renal failure

## Abstract

**Introduction:**

Acute kidney injury (AKI) affects ~30% of hospitalized neonates. Critical to advancing our understanding of neonatal AKI is collaborative research among neonatologists and nephrologists. The Neonatal Kidney Collaborative (NKC) is an international, multidisciplinary group dedicated to investigating neonatal AKI. The AWAKEN study (Assessment of Worldwide Acute Kidney injury Epidemiology in Neonates) was designed to describe the epidemiology of neonatal AKI, validate the definition of neonatal AKI, identify primary risk factors for neonatal AKI, and investigate the contribution of fluid management to AKI events and short-term outcomes.

**Methods and analysis:**

The NKC was established with at least one pediatric nephrologist and neonatologist from 24 institutions in 4 countries (USA, Canada, Australia, and India). A Steering Committee and four subcommittees were created. The database subcommittee oversaw the development of the web-based database (MediData Rave™) that captured all NICU admissions from 1/1/14 to 3/31/14. Inclusion and exclusion criteria were applied to eliminate neonates with a low likelihood of AKI. Data collection included: (1) baseline demographic information; (2) daily physiologic parameters and care received during the first week of life; (3) weekly “snapshots”; (4) discharge information including growth parameters, final diagnoses, discharge medications, and need for renal replacement therapy; and (5) all serum creatinine values.

**Ethics and dissemination:**

AWAKEN was proposed as human subjects research. The study design allowed for a waiver of informed consent/parental permission. NKC investigators will disseminate data through peer-reviewed publications and educational conferences.

**Discussion:**

The purpose of this publication is to describe the formation of the NKC, the establishment of the AWAKEN cohort and database, future directions, and a few “lessons learned.” The AWAKEN database includes ~325 unique variables and >4 million discrete data points. AWAKEN will be the largest, most inclusive neonatal AKI study to date. In addition to validating the neonatal AKI definition and identifying risk factors for AKI, this study will uncover variations in practice patterns related to fluid provision, renal function monitoring, and involvement of pediatric nephrologists during hospitalization. The AWAKEN study will position the NKC to achieve the long-term goal of improving the lives, health, and well-being of newborns at risk for kidney disease.

## Introduction

Over the last decade, the nephrology and critical care communities have repeatedly shown that acute kidney injury (AKI) in children (1, 2) and adults (3–7) portends poor short-term and long-term outcomes independent of severity of illness. The available short-term outcome data in neonates are similar: neonates with AKI have increased rates of mortality and longer hospital stays as compared to those without AKI (Table 1) (8–14). The prevalence of AKI reaches ~30% in neonates admitted to a tertiary level neonatal intensive care unit (NICU) (8–13, 14). However, prevalence estimates and data on short-term outcomes are solely derived from small retrospective, single-center studies. Moreover, little is known about the long-term consequences of neonatal AKI (15). Many significant questions, including how to best define, risk factors for, incidence of, association with other co-morbidities, and the short-term and long-term outcomes after AKI remain unanswered.

**Table 1:** **Neonatal AKI prevalence and mortality rates**.

	Prevalence (%)	Mortality AKI vs. no AKI (%)	Reference
VLBW^a^	18	55 vs. 5	(8, 14)
	40	14 vs. 4	
ELBW^b^	13	70 vs. 22	(9)
Sick near term/term	18	22 vs. 0	(10)
Sepsis	26	70 vs. 25	(13)
Asphyxiated	38	14 vs. 2	(12)
ECMO	71	73 vs. 20^c^	(11)

*^a^Very low birth weight (VLBW) infants <1500 g*.

*^b^Extremely low birth weight (ELBW) infants <1000 g*.

*^c^Extracorporeal membrane oxygenation (ECMO)*.

Critical to advancing the field of neonatal AKI is collaborative research among neonatologists and pediatric nephrologists. To foster this partnership, the National Institutes of Health (NIH) sponsored a multidisciplinary workshop on neonatal AKI in April 2013 (16, 17). Soon after, the Neonatal Kidney Collaborative (NKC) was formed to address the critical gaps in knowledge highlighted at the workshop. The NKC is an international working group composed of neonatologists and pediatric nephrologists committed to advancing the field of neonatal AKI research. The first mission of the NKC was to develop the infrastructure to function. The second mission of the NKC was the development of the AWAKEN study (Assessment of Worldwide Acute Kidney injury Epidemiology in Neonates), a large, retrospective cohort study designed with the following aims: (1) determine if the Kidney Disease: Improving Global Outcomes (KDIGO) AKI definition, adapted to neonates, is independently associated with mortality, length of stay, and discharge serum creatinine (sCr), (2) define the risk factors associated with neonatal AKI, (3) determine how fluid balance during the first few weeks of life relates to biochemical data and clinical outcomes, and (4) assess the performance of different definitions of AKI in neonates.

Assessment of Worldwide Acute Kidney injury Epidemiology in Neonates is the largest and most inclusive neonatal AKI study to date. Evaluation of these important questions will improve care and stimulate new research questions that will drive this field for many years. Collaborations will foster strong relationships between neonatologists and pediatric nephrologists within and across centers. The infrastructure created to perform the AWAKEN study will position this group to conduct additional clinically relevant, hypothesis-driven studies aimed at improving the lives, health and well-being of patients at risk for kidney disease as the result of events during the newborn period. Here, we describe the formation of the NKC, the establishment of the AWAKEN cohort and database, future directions, and a few “lessons learned” in the process.

### Development of the Neonatal Kidney Collaborative

The NKC is a voluntary, non-funded group made up of pediatric centers who agreed to ensure: (1) the participation of both a neonatologist and pediatric nephrologist from their institution and (2) local IRB approval. The NKC membership evolved from professional relationships developed through previous clinical and research interactions including the 2013 NIH workshop “Neonatal AKI.” A current roster of participating institutions is included in Table 2.

**Table 2:** **Participating institutions**.

Institution	Location (city, state, country)	Enrolled in AWAKEN (*n*)	% inborn	Creatinine assay
Canberra Hospital	Canberra, ACT, Australia	37	76	Jaffe
Children’s Hospital Colorado	Denver, CO, USA	68	7	Enzymatic
Children’s Hospital at Montefiore	Bronx, NY, USA	91	96	Enzymatic
Children’s National Medical Center	Washington, DC, USA	86	1	Enzymatic
Cincinnati Children’s Hospital Medical Center	Cincinnati, OH, USA	81	9	Enzymatic
Maimonides Medical Center	Brooklyn, NY, USA	53	94	Enzymatic
Medanta –The Medicity Hospital	Gurgaon Haryana, India	58	22	Jaffe
MetroHealth Medical Center	Cleveland, OH, USA	69	100	Jaffe
Montreal Children’s Hospital/McGill University	Montreal, Quebec, Canada	67	0	Jaffe
Nationwide Children’s Hospital	Columbus, OH, USA	81	0	Enzymatic
Stony Brook Children’s Hospital	Stony Brook, NY, USA	78	90	Enzymatic
Texas Children’s Hospital	Houston, TX, USA	106	67	Enzymatic
Tufts University	Boston, MA, USA	87	67	Enzymatic
University of Alabama at Birmingham	Birmingham, AL, USA	59	63	Jaffe
University of British Columbia and Children’s and Women’s Health Center of British Columbia Branch	Vancouver, BC, Canada	104	63	Enzymatic
University of Iowa	Iowa City, IO, USA	121	58	Enzymatic
University of Kentucky	Lexington, KY, USA	116	54	Enzymatic
University of Miami	Miami, FL, USA	192	90	Enzymatic
University of Michigan	Ann Arbor, MI, USA	93	72	Jaffe
University of New Mexico	Albuquerque, NM, USA	81	81	Enzymatic
University of Rochester	Rochester, NY, USA	151	77	Enzymatic
University of Virginia	Charlottesville, VA, USA	101	66	Jaffe
University of Washington	Seattle, WA, USA	63	98	Both
Washington University in St. Louis	St. Louis, MO, USA	120	1	Enzymatic

**Figure 1:**
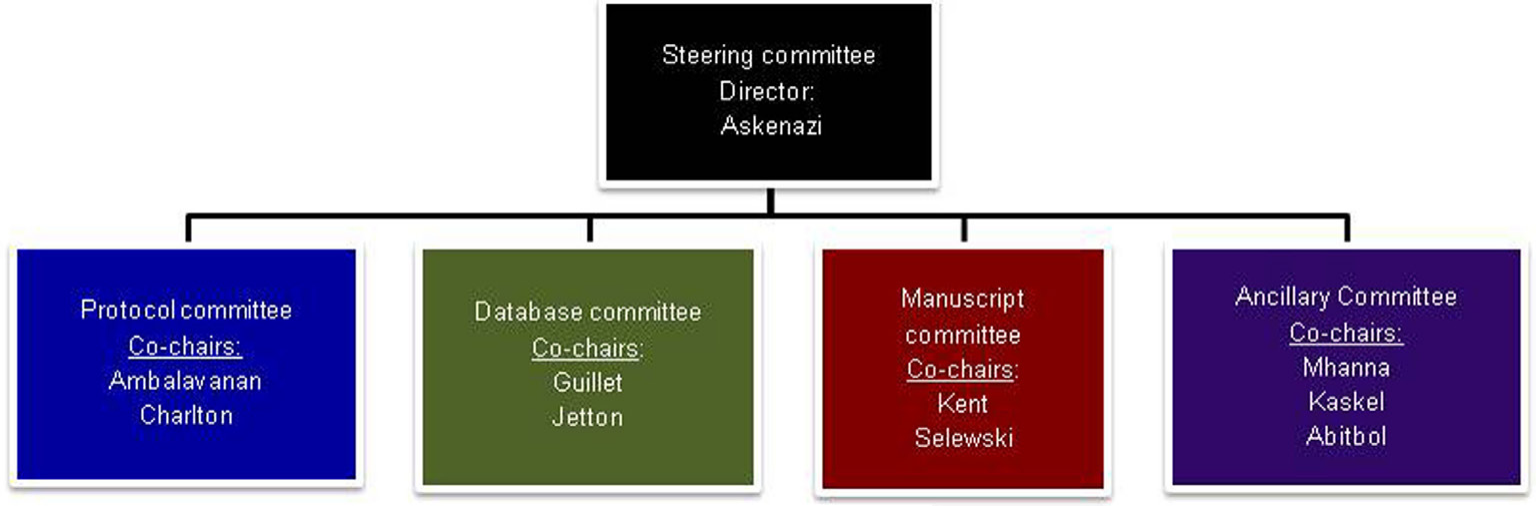
**The Steering committee is composed of the director and the co-chairs of each of the sub-committees**. The Protocol Committee was tasked to provide oversight and critique of the protocols submitted to the committee, both for the initial retrospective study (AWAKEN) and any future studies. It will also be charged with creating and submitting protocols to funding agencies and Institutional Review Boards. Other responsibilities include establishing rules for Primary Investigator designation and a system for group involvement for the establishment of future protocols. The Database Committee was charged with the development of the database, including the Manual of Procedures and Case Report Forms. Input was solicited from the NKC membership as to the data needed to answer the specific questions of interest for the AWAKEN study. These suggestions were collated and presented to the Steering Committee for final review. The myriad of data potentially available and the number of questions to be answered had the potential for an unwieldy and overwhelming amount of information. Data points that were included were thoroughly vetted by both nephrologists and neonatologists to balance the time of data collection with quality of the data elements. Once agreement was reached, in concert with the Data Management Center at Cincinnati Children’s Hospital Medical Center, electronic data forms were developed, tested, and finalized. The Manuscript Committee is responsible for initiating and developing abstracts for national and international meetings and manuscripts for submission to peer-reviewed journals. This committee will also review these abstracts and manuscripts prior to submission and provide the authors suggestions, as well as determine their suitability. The Ancillary Studies Committee will be responsible for developing rules on requesting use of data for ancillary studies and in developing these ideas into abstracts and manuscripts.

The NKC infrastructure consists of the Steering Committee overseen by the Director and the co-chairpersons of the four subcommittees (Figure 1). Each subcommittee (Protocol Committee, Manuscript Committee, Database Committee, and Ancillary Studies Committee) is co-chaired by a pediatric nephrologist and a neonatologist, thereby assuring that the clinical expertise of both specialties is represented.

### The AWAKEN Study

While research interest in neonatal AKI has increased greatly over the last 5 years, advances in clinical recognition, diagnosis, and supportive care for AKI in this population have lagged behind advances seen for adult and older pediatric patient groups (18). One major limitation to moving this field forward has been the lack of a standardized and validated definition of AKI in neonates similar to those used in adult and pediatric patients [e.g., risk, injury, failure, loss and end-stage RIFLE (19), KDIGO (20)]. The lack of a common definition has led to an inability to recognize AKI early and consistently across patients as well as an inability to pool or compare data across studies. In addition, the unique renal physiology of preterm and term infants creates challenges for the use of serum creatinine (sCr) as an AKI biomarker in these patients. Neonatal SCr initially reflects maternal values and then decreases over subsequent weeks after birth at different rates depending on gestational age. In addition, “normal” serum creatinine levels vary widely based on weight and gestational age. Before 2005, the majority of neonatal AKI studies used an arbitrary definition of AKI defined by a serum creatinine concentration (SCr) ≥1.5 mg/dl. In 2012, Jetton and Askenazi (21) proposed a standardized neonatal AKI definition based on the KDIGO definition for adults and children (Table 3) that identifies three levels of AKI severity based on graded changes in both serum creatinine and urine output. This and similar definitions have been utilized successfully in select neonatal patient populations (8, 10–12, 14, 22, 23). Consensus at the NIH neonatal AKI workshop was that this definition, despite its limitations, was currently the most appropriate definition to adopt and validate (Michael Zappetelli, personal communication). The group stressed the need to conduct large multi-center studies to test the performance of this definition against clinically relevant outcomes and further refine the definition based on data generated from these large cohorts. Thus, the AWAKEN study was designed to fulfill this charge and determine the extent to which the definition of AKI is associated with the short-term outcomes of mortality, length of stay, and discharge sCr. Although the association of neonatal AKI with longer term outcomes is beyond the scope of the AWAKEN study, the data generated will provide the foundation for prospective follow-up studies and the groundwork for prospective studies of at-risk populations.

**Table 3:** **Neonatal acute kidney injury KDIGO classification (21)**.

Stage	Serum creatinine	Urine output
0	No change in sCr *or* rise <0.3 mg/dl	> 1 ml/kg/h
1	sCr rise ≥0.3 mg/dl within 48 h or sCr rise ≥1.5–1.9× reference sCr^a^ within 7 days	> 0.5 ml/kg/h and ≤ 1 ml/kg/h
2	sCr rise ≥2–2.9× reference sCr^a^	>0.3 ml/kg/h and ≤ 0.5 ml/kg/h
3	sCr rise ≥3× reference SCr^a^ or sCr ≥2.5 mg/dl^b^ or receipt of dialysis	≤ 0.3 ml/kg/h

Differences between the proposed neonatal AKI definition and KDIGO include:

*^a^Reference sCr will be defined as the lowest previous sCr value*.

*^b^sCr value of 2.5 mg/dl represents less than 10 ml/min/1.73m^2^*.

Similarly, fluid overload has been identified as a major risk factor for morbidity and mortality in adult and pediatric patients with AKI (24, 25), but has not been fully examined in the neonatal population. As with the study of sCr in this group, the unique physiology of neonates, especially premature and low-birth weight infants, creates challenges for applying concepts developed in older patients to this special group. In the first few days after birth, healthy term infants will lose around 7% of their weight. Depending on the amount of fluid provision, ambient temperature and humidity, gestational age, and kidney function, infants admitted to the NICU will have different amounts of weight losses due to changes in fluid balance over the first weeks of life (26).

Appropriate fluid balance is critical to the care of newborns. In premature infants, mild fluid weight losses over the first week of life are desirable as high fluid intake can be associated with patent ductus arteriosus, bronchopulmonary dysplasia, cardiac failure, necrotizing enterocolitis, and intraventricular hemorrhage (27). Alternatively, inadequate fluid provision may cause hemodynamic dysfunction potentially resulting in AKI. However, in the extremely low-birth weight (ELBW) and extremely low gestational age neonate (ELGAN), fluid balance cannot be calculated in the same way as in older, larger neonates, or pediatric patients [e.g., using documented intake and output volumes to calculate fluid overload or fluid deficits (28)]. Transepidermal fluid losses (TEFL) in the first week will vary considerably as a function of ambient humidity and are impossible to measure in clinical practice. Although at birth TEFL in healthy full-term neonates is similar to that in older children and adults (4–8 g/m^2^/h), it is estimated to be 75 g/m^2^/h in neonates born at 23 weeks’ gestation (29). Humidified incubators simplify the management of fluids in these infants and help prevent extremes of electrolyte abnormalities. An important objective in AWAKEN will be to determine if variations in body weight are an adequate surrogate for fluid balance in this population rather than the reliance on recorded intake and output for calculation of fluid overload as is used in older patients. We also seek to determine whether fluid overload functions as a sign of kidney injury and increases the risk of morbidity and mortality as has been shown in older patient groups.

The primary hypothesis of AWAKEN is that neonatal AKI is common and associated with increased mortality, longer length of NICU stay and higher discharge serum creatinine, even after controlling for severity of illness, interventions and demographics. The two secondary hypotheses of AWAKEN include (1) maternal and infant risk factors can predict AKI and (2) fluid balance is associated with clinical outcomes (Table 4). Ultimately, the NKC will be positioned to test and validate the proposed neonatal AKI definition and compare it with other AKI definitions to determine which definition is the most accurate. We anticipate that the AWAKEN study can validate these hypotheses because of the large sample size and rich data set. This multicenter study will also uncover variations in practice patterns among NICUs in relation to fluid provision, renal function monitoring, bedside identification of AKI and AKI risk factors, and the involvement of pediatric nephrologists during inpatient hospitalization. Moreover, AWAKEN will provide important information regarding critical time and data points necessary to build a meaningful prospective study in the future.

**Table 4:** **Primary hypotheses of AWAKEN**.

*Hypothesis #1*: Neonatal AKI is associated with short-term risk, even when adjusted for gestational age at birth, birthweight, 5 min Apgar score, congenital renal anomalies, and severity of illness score	Survival of infants to discharge or 120 days of age (or to 36 weeks’ postmenstrual age in infants born preterm) is less likely in babies with the diagnosis of AKI
	Length of stay is longer in infants with neonatal AKI
	Discharge serum creatinine is higher in infants with neonatal AKI
*Hypothesis #2*: Maternal and infant risk factors predict AKI	
*Hypothesis #3*: Fluid balance during the first week after birth is associated with short-term risk	Survival of infants to discharge or 120 days of age (or to 36 weeks’ postmenstrual age in infants born preterm) is less likely in babies with excessive fluid intake compared to output
	Changes in weight are a better indication of fluid balance, especially in the preterm population, than difference between fluid intake and measured output
	Length of stay is longer in infants with neonatal AKI
	Pulmonary outcomes, as measured by time to extubation and development of bronchopulmonary dysplasia, are worse in infants with evidence of fluid overload
	Discharge serum creatinine is higher in infants with fluid overload

## Methods and Analysis

### Design

**Figure 2:**
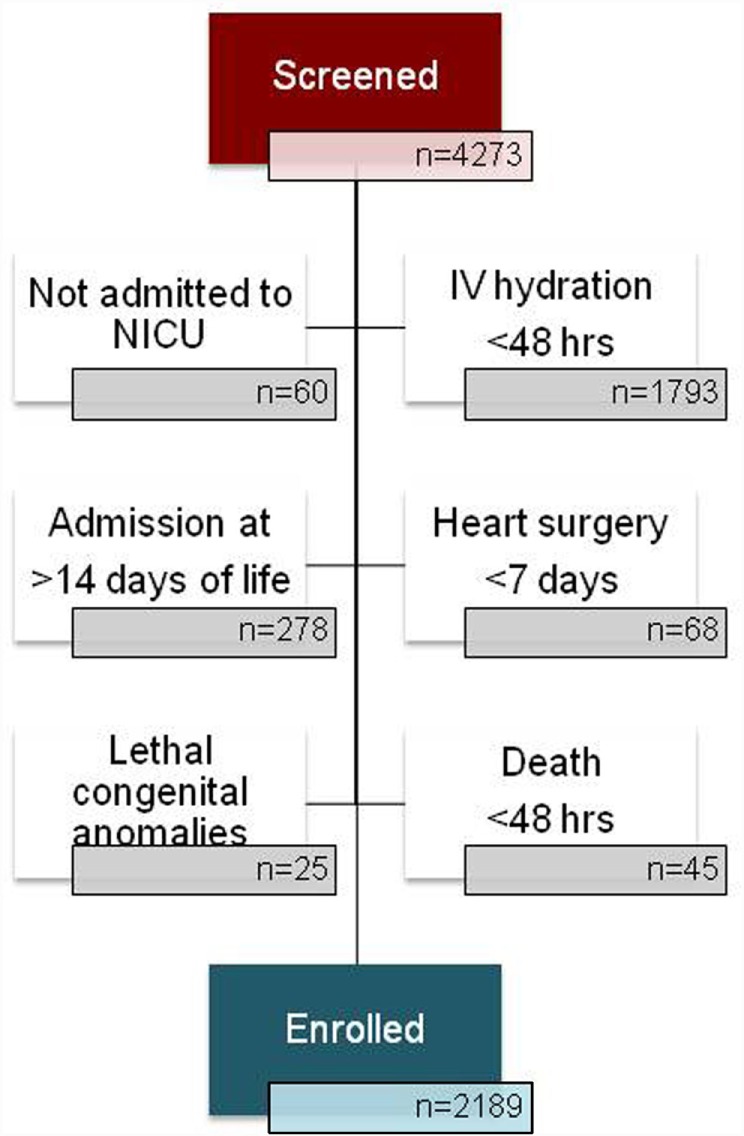
**Of the 4273 NICU admissions at the 24 participating institutions during the period 1/1/14–3/31/14, 2162 patients were enrolled**. The majority of those not included did not receive at least 48 h of IV hydration and/or nutrition. The second most common reason for exclusion was admission to the NICU at greater than 14 days of age. Subjects may have been excluded for more than one reason and may be counted more than once in the “not enrolled” numbers.

Assessment of Worldwide Acute Kidney injury Epidemiology in Neonates is a multi-center, international, retrospective cohort study. All neonates who fulfilled the inclusion and exclusion criteria at each of the 24 participating centers from January 1, 2014 until March 31, 2014 were enrolled (Figure 2). Detailed data collection continued until one of the following endpoints was reached: discharge to home, transfer to another facility or out of the NICU for escalating or convalescent care, death or 120 days of age. Limited information was collected between 120 days and final disposition. The study was registered at ClinicalTrials.gov (https://clinicaltrials.gov/ct2/show/NCT02443389?term=kidney+awaken&rank=1).

### Setting

Twenty-four level 2–4 NICUs in the United States (*n* = 20), Canada (*n* = 2), India (*n* = 1), and Australia (*n* = 1). A broad description of each institution is listed in Table 2, including the number of neonates enrolled in AWAKEN, the percentage of neonates born at that institution and the type of assay used to measure serum creatinine.

### Inclusion and Exclusion Criteria

All infants admitted from January 1, 2014 to March 31, 2014 who were ≤14 days of age and received at least 48 h of intravenous (IV) fluids as their primary source of hydration or nutrition (Table 5). Neonates who received IV fluids given solely for the administration of medications or line flushes were not included. The inclusion criteria were designed to capture sick neonates at significant risk for AKI and those who had an expected hospitalization of at least 48 h. Infants receiving routine care in the newborn nursery were not included in this study. The exclusion criteria were designed to omit patients with a limited hospitalization in the NICU or who had significant incomplete data particularly in the first 2 weeks of life. This exclusion is based on the belief that the first 2 weeks of life encompass a critical at-risk time for AKI. Because of the variable center-specific practice patterns regarding newborns with congenital heart disease requiring surgical repair (i.e., transfer to the PICU for post-operative care) and because these infants have been the subject of a number of previous AKI studies, neonates who required cardiac surgery within 7 days of birth were excluded. Infants with isolated ventricular septal defect, atrial septal defect, or patent ductus arteriosus were included unless they were transferred out of the NICU <7 days for surgical repair. Premature infants who remained in the NICU for ≥7 days to gain weight and grow in preparation for surgery were also included.

**Table 5:** **Inclusion and exclusion criteria**.

Inclusion criteria	Exclusion criteria
Admitted to participating NICU between 1/1/14 and 3/31/14 ≥48 h of IV fluids	Age greater than 14 days at admission Congenital heart disease with surgery <7 days of life Lethal chromosomal anomaly; including trisomy 13, 18 and anencephaly Neonatal mortality <48 h

All neonates admitted to the participating NICUs were screened for inclusion criteria. Demographic data including sex, self-reported ethnicity, race, date of birth, and date of NICU admission were collected for all neonates (Supplementary Materials – Data Sheet 1 – Screening Form). Date of birth was required as an “anchor” for the database format and the generation of the appropriate daily and weekly forms and was designated as “day 1.”

### Variable Collection

The AWAKEN study was designed to acquire information that is most likely related to kidney function and kidney injury in critically ill neonates. All sCr values during the study period were recorded. Data collection was otherwise organized into five components. Case report forms (CRF) and manual of operations are included in Supplementary Materials, Data Sheet 2.

#### Baseline Demographics

Maternal characteristics including age, gravid, parity, health conditions (chronic and pregnancy-associated), peri-partum infections, complications, and medications received; neonatal characteristics including site of delivery (inborn or outborn), mode of delivery, gestational age and birthweight, length and head circumference, initial temperature, resuscitation data, and reasons for admission.

#### Daily Information for Week 1

Weight, blood pressure and heart rate, highest level of respiratory support, fluid intake (intravenous and enteral fluids), fluid output (urine output and total), nephrotoxic medications, and laboratory parameters (hemoglobin, blood urea nitrogen, sodium, albumin, blood cultures, urine cultures, and cerebrospinal fluid cultures).

#### Weekly “Snapshots” for the Remaining Weeks of Data Collection

The first value of the week for all of the variables included in the daily form until the neonate reached the endpoint.

#### Discharge Data

Disposition of the infant at the end of the data collection period (“status” – discharged home prior to 120 days, still in the NICU at ≥120 days, transferred to another facility for convalescent care, transferred to another facility for escalation of care or died on or prior to 120 days), growth parameters, discharge medications if kidney related (urinary tract infection prophylaxis, antihypertensive medications and diuretics), and discharge diagnoses. Detailed information about kidney diagnoses (congenital abnormalities, episodes of AKI and need for nephrology consults) and renal replacement therapy provided were collected.

#### Prolonged Length of Stay

Date of final disposition and reason for prolonged NICU stay >120 days were collected, as were the highest and last creatinine obtained between 120 days and final disposition.

Because this is a retrospective study, all data reflect current, local standards of care. Definitions of terms listed in the Manual of Procedures were evidence-based whenever possible or consistent with those used in other published research studies.

#### MediData Rave™

Data entry of the variables of interest was performed by participating sites using a web-based database, MediData Rave™. Rave™ is a commercially available system designed to capture, manage, and report clinical research data. Through this system, each participating site was assigned a unique code by the database management team. If responses to the initial inclusion and exclusion criteria provided by the individual performing the data entry fulfilled study criteria, the system dynamically generated the remainder of the patient casebook, opening the “gateway” for the site to enter additional data for an enrolled patient. When eligibility was determined, the system guided the data entry personnel at each site to enter the clinical variables of interest. Limited protected health information (date of birth) was required to generate the proper dates for the daily and weekly forms. Release of the date of birth as part of the limited data set was agreed upon in the Data Use Agreement between each site and the primary study site. All participating sites used the same case report forms (CRFs). The electronic CRFs were designed, in partnership with the project’s Database Committee, by the representatives in the Cincinnati Children’s Hospital Medical Center Data Management Center (CCHMC DMC). Data entered by the sites were reviewed by the DMC. Values outside of expected ranges generate queries back to the individual site personnel. Query responses are then reviewed by the DMC personnel as well as physician members of the database subcommittee if clinical expertise was required to adjudicate the responses.

All site primary investigators and site personnel performing data entry completed commercially developed online training prior to being allowed access to MediData Rave™. Additional webinars sponsored by primary site personnel and Database Committee team were conducted to instruct sites on the proper use and interpretation of the Manual of Procedures (including definitions for study variables) and completion of CRFs. Co-chairs of the database committee and the lead coordinator were available for guidance and clarification as needed.

### Analyses

Data management and statistical analysis will be executed at the University of Alabama at Birmingham using SAS software v9.4 (SAS Institute, Cary, NC, USA). Analysis of data will be performed independently for each specific aim. Descriptive statistics will be used to describe the populations of NICU patients overall, with and without AKI. Data will be analyzed to compare the different definitions of neonatal AKI.

### Interventions

No interventions are included in this study protocol.

### Co-Enrollment

No conflict with any other ongoing studies as this is a retrospective observational study.

### Timeline

The MediData Rave™ database was piloted by centers from 1/17/15 to 1/28/15. Subsequently final adjustments of the manual of operation were made. Each institution was required to participate in database training on either 2/10/15 or 2/12/15. Additionally, each database entry personnel was required to participate in online training from 2/10/15 to 2/27/15. The database was open for data entry on 3/2/15. The trial was registered at ClinicalTrials.gov on 5/11/15. All institutions completed data entry by 12/12/15.

## Discussion

The AWAKEN study created the largest cohort of neonates assembled to date for exploring AKI in this vulnerable patient population. Across the 24 centers with high acuity NICUs distributed across 3 continents and 4 countries, we screened 4273 and enrolled 2162 newborns during the 3 months’ time-period. As demonstrated in Table 2, the institutions represented in the AWAKEN study include academic medical centers with diverse referral patterns, volumes of patients and creatinine assay methods. By collecting detailed demographic information on these babies, the NKC has created a rich database with which we will be able to describe the prevalence of neonatal AKI in different ethnic, racial, and geographic populations. Analyses can be performed based on birthweight, gestational age, and other criteria. Daily weights can be assessed as a surrogate for “fluid balance” in the first week after birth in ELBW neonates given the difficulty in accurately determining fluid status based solely on intake and measurable output. Although data points were chosen based on relatedness to renal function and injury, the database will be a source of carefully collected information on NICU patients that may be used to compare practices across sites and investigate various best practices in this population, including strategies for fluid provision.

The NKC represents an interdisciplinary group of clinicians and researchers interested in neonatal AKI with the unified goal of improving the lives, health and wellbeing of newborns at risk for kidney disease. Representation of institutions from four countries and three continents and the involvement of both pediatric nephrologists and neonatologists make the unique. Each subspecialty brings its expertise relevant to understanding the problem of neonatal renal development, function, and injury. For example neonatologists have less experience with the long-term ramifications of renal disorders seen in the newborn period, while pediatric nephrologists rarely are involved in the initial care of the extremely premature infants or those with severe perinatal asphyxia, including decisions regarding fluid provision and choice of drugs to treat or prevent various conditions. True collaborations such as this will allow a greater understanding of neonatal renal physiology and pathology and the impact on other organ systems. AWAKEN will provide the infrastructure and experience for gathering evidence-based data on best practices to improve short- and long-term kidney health in neonates.

The AWAKEN study does have several limitations, the most substantial being the retrospective design. The retrospective design will likely result in the absence of data points for many infants. Since the serum creatinine is ordered by the treating neonatologist who may be more likely to do so if there are clinical indicators for AKI, its incidence may be underestimated especially as AKI in neonates may be non-oliguric as in the case of gentamicin nephrotoxicity. However, we will gain insight into current practices for monitoring, diagnosing, and treating kidney function. By comparing practices between NICUs, we will acquire a sense of important time points for data collection for future prospective studies. In addition, we may be able to develop “best practices” standards of care for at-risk neonatal populations.

At the onset of the AWAKEN project the database committee, in conjunction with the Steering committee, scrutinized variables to address the aims of the study, with the goal of balancing the granularity of the data with the time commitment necessary for data extraction and entry. Even after limiting collection to those data points deemed crucial, the time required to complete screening and data entry for excluded patients was ~30 min and an average of 2 h for included patients, depending on the complexity of the medical conditions and length of stay.

As with many clinical studies, retrospective or prospective, much of the data extraction is done by trained research staff and not by physicians. We surveyed the participating centers and discovered the site principal investigator was responsible for a significant proportion of data extraction at only four institutions, and the data was entered by a physician at only two sites. In fact, since this was a study initiated without benefit of external funding, it is likely that the extent of direct physician involvement in the data extraction and entry was higher than if funding were available to hire additional research staff. Given the cross-disciplinary nature of the study, the familiarity of research staff with both neonatal and renal terminology, including definitions of diagnoses, may have been challenging. Several illustrative examples were encountered as the database was “cleaned” and queries generated. This unfamiliarity resulted in additional time and effort on the part of the center PI, the database committee and the data center personnel to ensure the integrity of the data prior to analysis. In future studies, more detail and training will be incorporated in the Manual of Procedures to address this issue.

Even with these limitations, NKC has an unprecedented opportunity to provide better estimates of the incidence and outcomes of neonatal AKI, and it is likely this work will help to refine the definition of neonatal AKI, improve our understanding of the risk factors associated with AKI, and raise awareness of AKI as an important clinical event during the care of critically ill neonates. We will improve our understanding of the contribution of fluid balance to biochemical and clinical outcomes. Additionally, by having a concise documentation of each infant’s hospital course, we will be able to infer the role which AKI and other potential risk factors have on the incidence of CKD in future studies. These collaborations, including the data and infrastructure, will enable the NKC to plan future intervention studies geared toward the reduction of AKI and improved short and long-term renal outcomes, including CKD.

## Ethics and Dissemination

### Ethics Approval and Consent to Participate

AWAKEN was proposed as human subjects research. The study design allowed for a waiver of informed consent/parental permission. Permission for limited protected health information (PHI) dataset was requested and granted by all sites, specifically to allow the use of date of birth for proper creation of the database as described above. The waiver of consent was pursued based on the following rationale:

- The research involves no more than minimal risk to the subjects.
- The waiver does not adversely affect the rights and welfare of the subjects.
- The research cannot practically be carried out without the waiver or alteration. This is a retrospective cohort study of infants admitted over a year prior to data collection. Requiring informed consent from every eligible patient would cause a significant reduction in enrollment and potentially introduce selection bias into the dataset.

The sites participating in AWAKEN obtained appropriate institutional review board approval from their respective centers and executed data use agreements with the data coordination center (Cincinnati Children’s Hospital Medical Center). Protocol amendments have been generated by the protocol committee and disseminated to each institution.

### Access to Data and Dissemination

Deidentified data is maintained at the University of Alabama at Birmingham. NKC investigators plan to disseminate data through peer-reviewed publications and through platform and poster presentations at educational conferences. The ancillary studies committee will provide data sets to NKC members following the approval of the project.

## Author Contributions

JGJ participated in the design and implementation of the study of the study, the preparation of the initial draft, and has critically reviewed the manuscript. RG participated in the design of the study and implementation of the study, the preparation of the initial draft, and has critically reviewed the manuscript. DA participated in the design and implementation of the study and has critically reviewed the manuscript. LD participated in the implementation of the study and has critically reviewed the manuscript. JJ participated in the design of this study, including the database programing and has critically reviewed the manuscript. AK participated in the design of the study and has critically reviewed the manuscript. DS participated in the design of the study and has critically reviewed the manuscript. CA participated in the implementation of the study and has critically reviewed the manuscript. FK participated in the implementation of the study and has critically reviewed the manuscript. MM participated in the implementation of the study and has critically reviewed the manuscript. NA participated in the implementation of the study and has critically reviewed the manuscript. JC participated in the design of the study, the preparation of the initial draft, and has critically reviewed the manuscript. All authors aided in the drafting of the work or revising of the work it critically for important intellectual content, approve the final version to be published, and agree to be accountable for all aspects of the work in ensuring that questions related to the accuracy or integrity of any part of the work were appropriately investigated and resolved.

## Conflict of Interest Statement

David Askenazi serves on the speaker board for BAXTER, and the AKI Foundation. Stuart L. Goldstein receives grant funding from Baxter Healthcare and is a consultant for Baxter, Bellco, Inc., Akebia, Inc., AM Pharma, Inc., and Astute Medical. Jonathan M. Klein is a consultant for Draeger Medical. Juan C. Kupferman is on the speaker’s Bureau and consultant for Alexion Pharmaceuticals. Subrata Sarkar is a consultant for GW Research Ltd, Cambridge, UK. The remaining authors declare that the research was conducted in the absence of any commercial or financial relationships that could be construed as a potential conflict of interest.

## Submitted on Behalf of Neonatal Kidney Collaborative

The following individuals served as collaborators and site investigators for the AWAKEN study. They participated in protocol development and review, local IRB submission, data collection and reviewed and revised the manuscript:

Ayse Akcan Arikan, Texas Children’s Hospital, Baylor College of Medicine, Houston, TX, USA; Alok Bhutada, Maimonides Medical Center, Brooklyn, New York, NY, USA; Elizabeth Bonachea, Nationwide Children’s Hospital, Columbus, OH, USA; Louis Boohaker, Children’s of Alabama, University of Alabama at Birmingham, Birmingham, AL, USA; Patrick D. Brophy, University of Iowa Children’s Hospital, Iowa City, IA, USA; Aftab S. Chishti, University of Kentucky, Lexington, KY, USA; Tarah T. Colaizy, University of Iowa Children’s Hospital, Iowa City, IA, USA; F. Sessions Cole, Washington University, St. Louis, MO, USA; Carl D’Angio, Golisano Children’s Hospital, University of Rochester, Rochester, New York, NY, USA; T. Keefe Davis, Washington University, St. Louis, MO, USA; Marissa DeFreitas, Holtz Children’s Hospital, University of Miami, Miami, FL, USA; Joshua Dower, Tufts University School of Medicine, Boston, MA, USA; Shahnaz Duara, Holtz Children’s Hospital, University of Miami, Miami, FL, USA; Jeffery Fletcher, Centenary Hospital for Women and Children, Canberra Hospital, Australian National University Medical School, Canberra, Australia; Mamta Fuloria, The Children’s Hospital at Montefiore, Bronx, New York, NY, USA; Jason Gien, University of Colorado, Children’s Hospital Colorado, Aurora, CO, USA; Katja M. Gist, University of Colorado, Children’s Hospital Colorado, Aurora, CO, USA; Stuart L. Goldstein, Cincinnati Children’s Hospital Medical Center, Cincinnati, OH, USA; Russell Griffin, Children’s of Alabama, University of Alabama at Birmingham, Birmingham, AL, USA; Mina H. Hanna, University of Kentucky, Lexington, KY, USA; Sangeeta Hingorani, University of Washington, Seattle Children’s Hospital, Seattle, WA, USA; Susan Ingraham, Nationwide Children’s Hospital, Columbus, OH, USA; Catherine Joseph, University of New Mexico Health Sciences Center, Albuquerque, NM, USA; Surender Khokhar, Apollo Cradle, Gurgaon, Haryana, India; Jonathan M. Klein, University of Iowa Children’s Hospital, Iowa City, IA, USA; Deepak Kumar, MetroHealth Medical Center, Case Western Reserve University, Cleveland, OH, USA; Juan C. Kupferman, Maimonides Medical Center, Brooklyn, New York, NY, USA; John Mahan, Nationwide Children’s Hospital, Columbus, OH, USA; Cherry Mammen, British Columbia Children’s Hospital, Vancouver, BC, Canada; Ayesa Mian, Golisano Children’s Hospital, University of Rochester, Rochester, New York, NY, USA; Lawrence Milner, Floating Hospital for Children at Tufts Medical Center, Tufts University School of Medicine, Boston, MA, USA; Arwa Nada, Nationwide Children’s Hospital, Columbus, OH, USA; Amy T. Nathan, Cincinnati Children’s Hospital Medical Center, Cincinnati, OH, USA; Robin Ohls, University of New Mexico Health Sciences Center, Albuquerque, NM, USA; Sofia Perazzo, Children’s National Medical Center, The George Washington University School of Medicine and the Health Sciences, Washington, DC, USA; Erin Rademacher, Golisano Children’s Hospital, University of Rochester, Rochester, New York, NY, USA; Rupesh Raina, MetroHealth Medical Center, Case Western Reserve University, Cleveland, OH, USA; Shantanu Rastogi, Maimonides Medical Center, Brooklyn, New York, NY, USA; Patricio E. Ray, Children’s National Medical Center, The George Washington University School of Medicine and the Health Sciences, Washington, DC, USA; Kimberly Reidy, The Children’s Hospital at Montefiore, Bronx, New York, NY, USA; Mary Revenis, Children’s National Medical Center, The George Washington University School of Medicine and the Health Sciences, Washington, DC, USA; Christopher J. Rhee, Texas Children’s Hospital, Baylor College of Medicine, Houston, TX, USA; Smriti Rohatgi, Medanta Kidney Institute, The Medicity Hospital, Gurgaon, Haryana, India; Subrata Sarkar, C.S. Mott Children’s Hospital, University of Michigan, Ann Arbor, MI, USA; Sidharth Kumar Sethi, Medanta Kidney Institute, The Medicity Hospital, Gurgaon, Haryana, India; Alexandra Smith, Floating Hospital for Children at Tufts Medical Center, Tufts University School of Medicine, Boston, MA, USA; Danielle E. Soranno, University of Colorado, Children’s Hospital Colorado, Aurora, CO, USA; Shanthy Sridhar, Stony Brook Children’s Hospital, Stony Brook, New York, NY, USA; Amy Staples, University of New Mexico Health Sciences Center, Albuquerque, NM, USA; Michelle Starr, University of Washington, Seattle Children’s Hospital, Seattle, WA, USA; Jonathan R. Swanson, University of Virginia Children’s Hospital, Charlottesville, VI, USA; Anne Synnes, British Columbia Children’s Hospital, Vancouver, BC, Canada; Sanjay Wazir, DM, Apollo Cradle, Gurgaon, Haryana, India; Pia Wintermark, Montreal Children’s Hospital, McGill University Health Centre, Montreal, QC, Canada; Craig S. Wong, University of New Mexico Health Sciences Center, Albuquerque, NM, USA; Robert Woroniecki, Stony Brook Children’s Hospital, Stony Brook, New York, NY, USA; Michael Zappitelli, Montreal Children’s Hospital, McGill University Health Centre, Montreal, QC, Canada.

## Abbreviations

AKI, acute kidney injury; AWAKEN, assessment of worldwide acute kidney injury epidemiology in neonates; CKD, chronic kidney disease; CRF, case report forms; CTSI, clinical and translational science institute; ELBW, extremely low birth weight; ELGAN, extremely low gestational age neonate; KDIGO, kidney disease: improving global outcomes; NICU, neonatal intensive care unit; NIH, National Institutes of Health; NKC, neonatal kidney collaborative; RIFLE, risk, injury, failure, loss- and end-stage; SCr, serum creatinine; TEFL, transepidermal fluid losses.
