# Supplementary material for: Assessment of Worldwide Acute Kidney Injury Epidemiology in Neonates: Design of a Retrospective Cohort Study
Source: Front Pediatr. 2016 Jul 19;4:68. doi: 10.3389/fped.2016.00068 (PMC4950470; doi:10.3389/fped.2016.00068)
Supplement: Supplementary file 1 [file Data_Sheet1.PDF]

Site subject number _____
Database subject number _____

SCREENING AND INTAKE FORM

Demographics

1. Gender __________
    a. male,
    b. female,
    c. ambiguous
2. Ethnicity ___________
    a. Hispanic/Latino/Spanish origin;
    b. Non-Hispanic/non-Latino/non-Spanish origin;
    c. unknown
3. Race - choose all that apply ___________
    a. White/Caucasian
    b. Black/African-American
    c. American Indian/Alaska native;
    d. Asian;
    e. Native Hawaiian or other Pacific Islander;
    f. unknown
    g. other
4. Date of birth ___________
5. Date of NICU admission ___________

Inclusion/Exclusion Criteria

Eligibility – must indicate yes to both 1 and 2.

- Inclusion criterion:
    1. All infants admitted to a participating center NICU (level 2 or 3) who do not meet exclusionary criteria

       Yes □
       No □

    2. Infants who receive at least 48 hours of intravenous hydration. This includes iv fluids to provide hydration and/or nutrition and does not include iv fluids solely for administration of medications.
       Yes □
       No □

- Exclusion criteria – <u>any</u> <u>one</u> will exclude patient
    1. Admission to the NICU at ≥ 14 days of age

       Yes □
       No □

Initials of the person completing this form __________________
Date completed ____

Site subject number _____
Database subject number _____

2. Congenital heart disease requiring surgery within the first 7 days

Yes □
No □

3. Lethal chromosomal anomalies

Yes □
No □

4. Infants who die at < 48 hours of age

Yes □
No □

Initials of the person completing this form ___________________
Date completed ____

Site subject number _____
Database subject number ______

BASELINE FORM

Maternal information:

- Maternal age at delivery in years ____________
- Gravida ____________
- Parity ____________

Maternal conditions – check all that apply

- ◻ No complications
- ◻ Maternal infections at or near the time of delivery including bacterial and viral infections.
  - ◻ Intrapartum bacterial
  - ◻ Intrapartum viral infection
- ◻ Maternal diabetes
- ◻ Maternal hypothyroidism
- ◻ Maternal chronic hypertension
- ◻ Maternal history of kidney disease
- ◻ Maternal pre-eclampsia
- ◻ Maternal eclampsia
- ◻ IUGR
- ◻ Oligohydramnios
- ◻ Polyhydramnios
- ◻ Maternal hemorrhage
- ◻ Multiple gestation,
  - ◻ all fetuses survived to birth
  - ◻ demise of one or more fetuses
  - ◻ no information is available about other fetuses
  - ◻ twin-twin transfusion
- ◻ Unknown
- ◻ Drugs documented used during this pregnancy – check all that apply
  - ◻ None
  - ◻ Maternal steroids for fetal maturation
  - ◻ ACE-inhibitors (captopril, enalapril, lisinopril, benazepril, fosinopril, quinapril, enalaprilat),
  - ◻ NSAIDs (including aspirin, ibuprofen, paracetamol, other over-the-counter pain relievers),
  - ◻ beta blockers (propranolol, atenolol, carvedilol, metoprolol, esmolol, labetalol),
  - ◻ calcium channel blockers (amlodipine, felodipine, isradipine, nicardipine),
  - ◻ vasodilators (hydralazine, minoxidil, nitroprusside),
  - ◻ central alpha-agonists (clonidine),
  - ◻ indomethacin (when given intrapartum for tocolysis),

Initials of person completing this form ____________
Date completed ___________________

- ◻ <u>illicit drugs</u> (such as cocaine, heroin, THC, and any other street drugs; also include drugs used in drug treatment programs such as methadone, subutex) by history or drug screening,
- ◻ <u>tobacco</u>,
- ◻ <u>alcohol</u>,
- ◻ <u>SSRIs</u> (citalopram/Celexa®, escitalopram/Lexapro®, fluvoxamine/Luvox®, paroxetine/Paxil®, fluoxetine/Prozac, ® sertraline/Zoloft®).
- ◻ <u>Heparin</u>
- ◻ <u>Warfarin</u>
- ◻ unknown

- ◻ Assisted conception
  - ◻ Yes
  - ◻ No
  - ◻ unknown

- ◻ Intrapartum complications – check all that apply
  - ◻ none
  - ◻ nuchal cord
  - ◻ meconium
  - ◻ severe maternal vaginal bleeding
  - ◻ cord rupture
  - ◻ shoulder dystocia
  - ◻ unknown

Site subject number _____
Database subject number ______

Neonatal information

- Site of delivery
  - ◻ Inborn
  - ◻ Outborn
- Gestational age at birth — weeks __________ days __________
- Birthweight, grams __________
  - ◻ If outborn, admission weight (grams) __________
- Length , centimeters __________
  - ◻ If outborn, admission length (cms) __________
- Head circumference, centimeters __________
  - ◻ If outborn, admission head circumference (cms) __________
- Admission temperature (°C): __________
  - ◻ None available

- Mode of delivery

◻ vaginal, vertex
◻ vaginal, breech
◻ vaginal, unknown presentation
◻ scheduled C-section (no labor)
◻ unscheduled C-section
◻ C-section, no other details known
◻ unknown delivery mode

Resuscitation

- Apgar scores, enter all that are documented
  - ◻ 1 minute __________
  - ◻ 5 minute __________
  - ◻ 10 minute __________
  - ◻ 15 minute __________
  - ◻ 20 minute __________
- Cord blood gas results
  - ◻ arterial pH __________
  - ◻ arterial base excess __________
  - ◻ venous pH __________
  - ◻ venous base excess __________
  - ◻ vessel unspecified pH __________
  - ◻ vessel unspecified base excess __________
  - ◻ none available
- If no cord blood gases are available, enter a blood gas obtained during the first hour, if available.
  - ◻ pH __________
  - ◻ base excess __________
  - ◻ none available __________

Initials of person completing this form ____________
Date completed ___________________

- Resuscitation provided to the infant in the delivery room , check all that apply
    - □ none (aside from drying and stimulation)
    - □ supplemental oxygen
    - □ PPV (positive pressure)
    - □ intubation
    - □ chest compressions
    - □ epinephrine
    - □ normal saline
    - □ blood transfusion (whole blood or red blood cells)
    - □ unknown

- Reason for admission

    Enter the reason(s) for admission from the list provided.- check all that apply
    - □ "prematurity" if gestational age at birth is < 35 weeks;
    - □ Respiratory symptoms (requiring observation and close monitoring and support no greater than supplemental oxygen via oxyhood or low flow nasal cannula, < 2L/min), all diagnoses
    - □ Respiratory failure (continued need for respiratory support including conventional ventilation, high frequency ventilation, non-invasive ventilation, CPAP, or High Flow Nasal Cannula ≥ 2L/min), all diagnoses
    - □ Sepsis evaluation
    - □ HIE (Hypoxic ischemic encephalopathy, birth asphyxia; 5-minute Apgar score < 6; initial pH < 7.0)
    - □ Seizures (can be clinical or electrographic or both)
    - □ Hypoglycemia (blood glucose < 35)
    - □ Hyperbilirubinemia (including need for phototherapy or exchange transfusion)
    - □ Dehydration
    - □ Metabolic evaluation (inborn error of metabolism, etc.)
    - □ Chromosomal anomaly (indicate if trisomy 21 or other chromosomal anomaly, unspecified)
    - □ Congenital heart disease
    - □ NEC
    - □ Omphalocele, Gastroschisis
    - □ Other surgical evaluation
    - □ Meningomyelocele
    - □ Other intracranial abnormalities
    - □ SGA (< 3%ile)
    - □ Other /specify____

- Length of time baby was in the NICU on the day of admission hours ___________
  mins ___________

Initials of person completing this form _____________
Date completed ____________________

Site subject number ______
Database subject number ______

WEEK ONE DATA

PLEASE NOTE: DAY OF BIRTH = DAY 1

Physiologic parameters: Please enter the highest, lowest and value closest to midnight (first)

	Day 1	Day 2	Day 3	Day 4	Day 5	Day 6	Day 7
date							
Weight (g)							
Systolic BP							
Highest							
Lowest							
First							
Diastolic BP							
Highest							
Lowest							
First							
Mean Arterial BP							
Highest							
Lowest							
First							

Initials of person completing the form
Date completed

Site subject number ______
Database subject number ______

Respiratory parameters:

1 ECMO

2 High frequency ventilation

3 Conventional ventilation

4 Noninvasive ventilation

5 CPAP

6 Nasal cannula

7 Oxyhood

8 No respiratory support

	Day 1	Day 2	Day 3	Day 4	Day 5	Day 6	Day 7
date							
Mode							
Max mean airway pressure							
Max FiO2							

Initials of person completing the form
Date completed

Site subject number _______
Database subject number _______

Fluid balance:

Fluid IN

Day	date	Quantifiable IV fluids<br>No=0<br>Yes=1	If yes,<br>IV fluid volume	Quantifiable enteral fluids<br>No=0<br>Yes=1	If yes,<br>Enteral fluid volume
1					
2					
3					
4					
5					
6					
7					

Fluid Out

Day	date	Was there fluid out in 24 hour period?<br>No = 0<br>Yes = 1	Quantifiable?<br>No=0<br>Yes=1	If yes,<br>Total volume
1				
2				
3				
4				
5				
6				
7				

Urine output

Day	date	Was there urine out in 24 hour period?<br>No = 0<br>Yes = 1	Quantifiable?<br>No=0<br>Yes=1	If yes,<br>Total volume
1				
2				
3				
4				
5				
6				
7				

Initials of person completing the form
Date completed

Site subject number ______
Database subject number ______

Medications: "0" = no; "1" = yes for any part of that day

	Day 1	Day 2	Day 3	Day 4	Day 5	Day 6	Day 7
date							
Aminoglycoside							
Vancomycin							
Piperacillin-Tazobactam							
Amphotericin							
Acyclovir							
Indomethacin							
ibuprofen							
hydralazine							
ACE-inhibitors							
Dopamine							
Dobutamine							
Milrinone							
Epinephrine							
Norepinephrine							
Furosemide							
Bumetanide							
Chlorothiazide							
Ethacrynic acid							
Spironolactone							
Theophylline							
Caffeine							

Initials of person completing the form
Date completed

Site subject number ______
Database subject number ______

Laboratory values: Include "worst" for day if more than one value obtained (highest creatinine, highest BUN, lowest albumin, lowest hemoglobin or hematocrit, highest and lowest sodium)

	Day 1	Day 2	Day 3	Day 4	Day 5	Day 6	Day 7
date							
BUN							
Albumin							
Hemoglobin							
Hematocrit							
Sodium							
Highest							
Lowest							
Blood culture<br>0 = negative<br>1 = positive<br>2 = not done							
CSF culture<br>0 = negative<br>1 = positive<br>2 = not done							
Urine culture<br>0 = negative<br>1 = positive<br>2 = not done							

Initials of person completing the form
Date completed

Site subject number ________
Database subject number ________

WEEKLY DATA

Physiologic parameters: Please enter the highest, lowest and value closest to midnight for **the day closest to the first day of each week (day 8, 15, 22, 29, etc.)**

	Week 2	Week 3	Week 4	Week 5	Week 6	Week 7	Week 8
date							
Weight (g)							
Systolic BP							
Highest							
Lowest							
First							
Diastolic BP							
Highest							
Lowest							
First							
Mean arterial pressure							
Highest							
Lowest							
First							
	Week 9	Week 10	Week 11	Week 12	Week 13	Week 14	Week 15
date							
Weight (g)							
Systolic BP							
Highest							
Lowest							
First							
Diastolic BP							
Highest							
Lowest							
First							
Mean arterial pressure							
Highest							
Lowest							
First							
	Week 16	Week 17	Week 18				
date							
Weight (g)							
Systolic BP							
Highest							
Lowest							
First							
Diastolic BP							
Highest							

Initials of person completing form ________________
Date completed _________________

Site subject number ________
Database subject number ________

Lowest							
First							
Mean arterial pressure							
Highest							
Lowest							
First							

Initials of person completing form _________________
Date completed __________________

Site subject number ________
Database subject number ________

Respiratory parameters:

1 ECMO
2 High frequency ventilation
3 Conventional ventilation
4 Noninvasive ventilation
5 CPAP
6 Nasal cannula
7 Oxyhood
8 No respiratory support

	Week 2	Week 3	Week 4	Week 5	Week 6	Week 7	Week 8
date							
Mode							
Max mean airway pressure							
Max FiO2							
	Week 9	Week 10	Week 11	Week 12	Week 13	Week 14	Week 15
date							
Mode							
Max mean airway pressure							
Max FiO2							
	Week 16	Week 17	Week 18				
date							
Mode							
Max mean airway pressure							
Max FiO2							

Initials of person completing form ________________
Date completed _________________

Site subject number ________
Database subject number ________

Fluid balance: Enter intake/output **for first day of each week (day 8, 15, 22, 29, etc)**

Intake

Week	Date	Quantifiable IV fluids? No=0 Yes=1	Total IV fluids volume	Quantifiable Enteral fluids? No=0 Yes=1	Total enteral fluid volume
2					
3					
4					
5					
6					
7					
8					
9					
10					
11					
12					
13					
14					
15					
16					
17					
18					

Total fluid output (urine plus other)

Week	Date	Was there fluid output? No = 0 Yes = 1	Quantifiable? No=0 Yes=1	Total volume
2				
3				
4				
5				
6				
7				
8				
9				
10				
11				
12				
13				
14				
15				
16				
17				

Initials of person completing form ________________
Date completed _________________

Site subject number ________
Database subject number ________

18				

Initials of person completing form ________________
Date completed _________________

Site subject number ________
Database subject number ________

<u>Medications</u>: "0" = no; "1" = yes **<u>for the first day of each week</u>**

	Week 2	Week 3	Week 4	Week 5	Week 6	Week 7
date						
Aminoglycoside						
Vancomycin						
Piperacillin-Tazobactam						
Amphotericin						
Acyclovir						
Indomethacin						
ibuprofen						
hydralazine						
ACE-inhibitors						
Dopamine						
Dobutamine						
Milrinone						
Epinephrine						
Norepinephrine						
Furosemide						
Bumetanide						
Chlorothiazide						
Ethacrynic acid						
Spironolactone						
Theophylline						
Caffeine						
	Week 8	Week 9	Week 10	Week 11	Week 12	Week 13
date						
Aminoglycoside						
Vancomycin						
Piperacillin-Tazobactam						
Amphotericin						
Acyclovir						
Indomethacin						
Ibuprofen						
hydralazine						
ACE-inhibitors						
Dopamine						
Dobutamine						
Milrinone						

Initials of person completing form ________________
Date completed _________________

Site subject number ________
Database subject number ________

Epinephrine						
Norepinephrine						
Furosemide						
Bumetanide						
Chlorothiazide						
Ethacrynic acid						
Spironolactone						
Theophylline						
Caffeine						
	Week 14	Week 15	Week 16	Week 17	Week 18	
date						
Aminoglycoside						
Vancomycin						
Piperacillin-Tazobactam						
Amphotericin						
Acyclovir						
Indomethacin						
Ibuprofen						
hydralazine						
ACE-inhibitors						
Dopamine						
Dobutamine						
Milrinone						
Epinephrine						
Norepinephrine						
Furosemide						
Bumetanide						
Chlorothiazide						
Ethacrynic acid						
Spironolactone						
Theophylline						
Caffeine						

Initials of person completing form ________________
Date completed _________________

Site subject number ________
Database subject number ________

Laboratory values: Include "worst" **for first day of each week** if more than one value obtained (highest creatinine, highest BUN, lowest albumin, lowest hemoglobin/hematocrit, highest and lowest sodium)

	Week 2	Week 3	Week 4	Week 5	Week 6	Week 7
Date						
BUN						
Albumin						
Hemoglobin						
Hematocrit						
Sodium						
Highest						
Lowest						
Blood culture<br>0 = negative<br>1 = positive<br>2 = not done						
CSF culture<br>0 = negative<br>1 = positive<br>2 = not done						
Urine culture<br>0 = negative<br>1 = positive<br>2 = not done						
	Week 8	Week 9	Week 10	Week 11	Week 12	Week 13
Date						
BUN						
Albumin						
Hemoglobin or hematocrit						
Sodium						
Highest						
Lowest						
Blood culture<br>0 = negative<br>1 = positive<br>2 = not done						
CSF culture<br>0 = negative<br>1 = positive<br>2 = not done						
Urine culture<br>0 = negative<br>1 = positive						

Initials of person completing form ________________
Date completed _________________

Site subject number ________
Database subject number ________

2 = not done						
	Week14	Week 15	Week 16	Week 17	Week 18	
Date						
BUN						
Albumin						
Hemoglobin or hematocrit						
Sodium						
Highest						
Lowest						
Blood culture<br>0 = negative<br>1 = positive<br>2 = not done						
CSF culture<br>0 = negative<br>1 = positive<br>2 = not done						
Urine culture<br>0 = negative<br>1 = positive<br>2 = not done						

Initials of person completing form ________________
Date completed _________________

Site subject number ________
Database subject number ________

Creatinine Values

Date	Time (if more than one creatinine level on the same date)	Value

Initials of person completing form _________________
Date completed __________________

Site subject number ________
Database subject number ________

Initials of person completing form ________________
Date completed _________________

Site subject number ______
Database subject number ______

DISCHARGE FORM

Disposition/"Status"

- □ Discharged home prior to120 days of age
- □ Still in NICU at $\geq$ 120 days of age
- □ Transferred to community hospital, other facility, or other hospital unit for convalescent care prior to 120 days
- □ Transferred to another hospital, facility or hospital unit, for escalation of care prior to 120 days
- □ Died in hospital at ≤ 120 days

Date of disposition/"status" __/__/____

Measurements at "status"

- Weight in grams _________
- Length in cms _________
- Head circumference in cms _________

Discharge medications

- □ antibiotics for urinary tract infection (UTI) prophylaxis
  - □ yes
  - □ no
- □ diuretics
  - □ yes
  - □ no
- □ antihypertensives:
  - □ yes
  - □ no

Initials of person completing the form ________________________
Date completed ____________________________

Site subject number ______
Database subject number ______

Discharge Diagnoses

- □ Cardiac
    - □ PDA confirmed
        - □ Self-resolved
        - □ treated with indomethacin only
        - □ surgical ligation with prior indomethacin treatment
        - □ surgical ligation without prior indomethacin treatment
    - □ Anatomic cardiac anomaly
    - □ Systemic hypertension
        - □ no medications at discharge
        - □ medications at discharge

- □ Pulmonary
    - □ BPD
        - □ requiring continued mechanical ventilation at 36 weeks' CGA
        - □ requiring continued non-invasive ventilation or CPAP at 36 weeks' CGA
        - □ requiring supplemental oxygen by nasal cannula or hood at 36 weeks' CGA
    - □ Persistent pulmonary hypertension confirmed
        - □ not requiring iNO or ECMO
        - □ requiring iNO
        - □ requiring ECMO

- □ Neurologic
    - □ IVH or PVL
        - □ None
        - □ IVH grade I

Initials of person completing the form ________________________
Date completed ____________________________

        - ☐ IVH grade II
        - ☐ IVH grade III
        - ☐ IVH grade IV
        - ☐ PVL (can be alone or in conjunction with IVH)
    - ☐ HIE (hypoxic ischemic encephalopathy/birth asphyxia)
    - ☐ Seizures
- ☐ GI
    - ☐ NEC
        - ☐ Bell Stage 2 – medically treated
        - ☐ Bell Stage 2 – surgically treated
    - ☐ Jaundice requiring an exchange transfusion
- ☐ Hematologic
    - ☐ DIC (disseminated intravascular coagulation)
- ☐ Infectious Disease
    - ☐ Culture negative sepsis (negative culture but treated with antibiotics for $\geq$ 5d)
    - ☐ Bacteremia
    - ☐ Viremia
    - ☐ Meningitis/encephalitis, include both bacterial and viral infections
- ☐ Metabolic abnormalities
- ☐ Endocrine abnormalities
- ☐ Genetic abnormalities
- ☐ Other major diagnoses
    - ☐ Specify___________________________________

Initials of person completing the form _________________________
Date completed _____________________________

Site subject number ______
Database subject number ______

Renal diagnoses

- ◻ Nephrology consult obtained during this admission.
- ◻ Acute kidney injury (coded) or acute renal failure coded. Includes ICD-9 codes 584.*
- ◻ Urinary tract infections. Please include only if there was a positive urine culture
- ◻ Medullary nephrocalcinosis/calcifications/kidney stones. Must be documented on renal ultrasound.

Congenital abnormalities of the kidney. (use most severe on Renal US or Discharge summary) Please circle all that apply from the list provided. See MOP for description

	Right	Left
Hypoplasia/Dysplasia	Yes NO	Yes NO
Multicystic Dysplastic kidney	Yes NO	Yes NO
Renal agenesis	Yes NO	Yes NO
Polycystic kidney disease		
	Yes, recessive	
	Yes, dominant	
	Unknown	
Horseshoe kidney	YES<br>NO	
Renal Ectopia	Yes NO	Yes NO
Hydronephrosis	Yes NO<br>Mild MOD Severe	Yes NO<br>Mild MOD Severe
UPJ	Yes NO	Yes NO
Hydroureter	Yes NO	Yes NO
Duplicated System	Yes NO	Yes NO
Posterior urethral valves	YES<br>NO	
Vesicoureteral reflux	Yes NO	Yes NO
	1 2 3 4 5	1 2 3 4 5
	Abnormality Present	
Urethral stricture	YES<br>NO	
Bladder exstrophy	YES<br>NO	
Neurogenic Bladder	YES<br>NO	
Prune Belly Syndrome	YES<br>NO	

Initials of person completing the form ________________________
Date completed ____________________________

<u>Renal replacement therapy</u>: *This section should be completed by the nephrologist member of your group.*

- ○ yes
- ○ no

- <u>If YES,</u>

  How many days did the patient receive any form of renal replacement therapy during the hospitalization? _______________

  ○ <u>Modality (please choose all that apply):</u>

  □ Peritoneal dialysis
  □ intermittent hemodialysis
  □ CRRT
  □ CRRT with ECMO
  □ SLED

  If CRRT (or CRRT with ECMO), Indicate modality

  □ *CVVH*
  □ *CVVHD*
  □ *CVVHDF*
  □ *SCUF*

  If CRRT Indicate machine (please choose all that apply)

  □ *Prismaflex*
  □ *NxStage*
  □ *Aquadex*
  □ *Fresenius*
  □ *In-line filter (for CRRT/ECMO only)*

  Type of anticoagulation (Only for ECMO with CRRT and CRRT alone)

  □ Heparin
  □ Citrate/calcium,
  □ None
  □ Unknown

Initials of person completing the form _______________________
Date completed ___________________________

Site subject number _____
Database subject number ______

BASELINE FORM

Maternal information:

- Maternal age at delivery in years ____________
- Gravida ____________
- Parity ____________

Maternal conditions – check all that apply

- ◻ No complications
- ◻ Maternal infections at or near the time of delivery including bacterial and viral infections.
    - ◻ Intrapartum bacterial
    - ◻ Intrapartum viral infection
- ◻ Maternal diabetes
- ◻ Maternal hypothyroidism
- ◻ Maternal chronic hypertension
- ◻ Maternal history of kidney disease
- ◻ Maternal pre-eclampsia
- ◻ Maternal eclampsia
- ◻ IUGR
- ◻ Oligohydramnios
- ◻ Polyhydramnios
- ◻ Maternal hemorrhage
- ◻ Multiple gestation,
    - ◻ all fetuses survived to birth
    - ◻ demise of one or more fetuses
    - ◻ no information is available about other fetuses
    - ◻ twin-twin transfusion
- ◻ Unknown
- ◻ Drugs documented used during this pregnancy – check all that apply
    - ◻ None
    - ◻ Maternal steroids for fetal maturation
    - ◻ ACE-inhibitors (captopril, enalapril, lisinopril, benazepril, fosinopril, quinapril, enalaprilat),
    - ◻ NSAIDs (including aspirin, ibuprofen, paracetamol, other over-the-counter pain relievers),
    - ◻ beta blockers (propranolol, atenolol, carvedilol, metoprolol, esmolol, labetalol),
    - ◻ calcium channel blockers (amlodipine, felodipine, isradipine, nicardipine),
    - ◻ vasodilators (hydralazine, minoxidil, nitroprusside),
    - ◻ central alpha-agonists (clonidine),
    - ◻ indomethacin (when given intrapartum for tocolysis),

Initials of person completing this form ____________
Date completed ____________________

- ◻ illicit drugs (such as cocaine, heroin, THC, and any other street drugs; also include drugs used in drug treatment programs such as methadone, subutex) by history or drug screening,
- ◻ tobacco,
- ◻ alcohol,
- ◻ SSRIs (citalopram/Celexa®, escitalopram/Lexapro®, fluvoxamine/Luvox®, paroxetine/Paxil®, fluoxetine/Prozac, ® sertraline/Zoloft®).
- ◻ Heparin
- ◻ Warfarin
- ◻ unknown

◻ Assisted conception
- ◻ Yes
- ◻ No
- ◻ unknown

◻ Intrapartum complications – check all that apply
- ◻ none
- ◻ nuchal cord
- ◻ meconium
- ◻ severe maternal vaginal bleeding
- ◻ cord rupture
- ◻ shoulder dystocia
- ◻ unknown

Site subject number _____
Database subject number ______

Neonatal information

- Site of delivery
  - ◻ Inborn
  - ◻ Outborn
- Gestational age at birth weeks __________
  days __________
- Birthweight, grams __________
  - ◻ If outborn, admission weight (grams) __________
- Length , centimeters __________
  - ◻ If outborn, admission length (cms) __________
- Head circumference, centimeters __________
  - ◻ If outborn, admission head circumference (cms) __________
- Admission temperature (°C): __________
  - ◻ None available

- Mode of delivery

◻ vaginal, vertex
◻ vaginal, breech
◻ vaginal, unknown presentation
◻ scheduled C-section (no labor)
◻ unscheduled C-section
◻ C-section, no other details known
◻ unknown delivery mode

Resuscitation

- Apgar scores, enter all that are documented
  - ◻ 1 minute __________
  - ◻ 5 minute __________
  - ◻ 10 minute __________
  - ◻ 15 minute __________
  - ◻ 20 minute __________
- Cord blood gas results
  - ◻ arterial pH __________
  - ◻ arterial base excess __________
  - ◻ venous pH __________
  - ◻ venous base excess __________
  - ◻ vessel unspecified pH __________
  - ◻ vessel unspecified base excess __________
  - ◻ none available
- If no cord blood gases are available, enter a blood gas obtained during the first hour, if available.
  - ◻ pH __________
  - ◻ base excess __________
  - ◻ none available __________

Initials of person completing this form ____________
Date completed ____________________

Site subject number _____
Database subject number ______

- Resuscitation provided to the infant in the delivery room , check all that apply
  - ◻ none (aside from drying and stimulation)
  - ◻ supplemental oxygen
  - ◻ PPV (positive pressure)
  - ◻ intubation
  - ◻ chest compressions
  - ◻ epinephrine
  - ◻ normal saline
  - ◻ blood transfusion (whole blood or red blood cells)
  - ◻ unknown

- Reason for admission

  Enter the reason(s) for admission from the list provided.- check all that apply
  - ◻ "prematurity" if gestational age at birth is < 35 weeks;
  - ◻ Respiratory symptoms (requiring observation and close monitoring and support no greater than supplemental oxygen via oxyhood or low flow nasal cannula, < 2L/min), all diagnoses
  - ◻ Respiratory failure (continued need for respiratory support including conventional ventilation, high frequency ventilation, non-invasive ventilation, CPAP, or High Flow Nasal Cannula ≥ 2L/min), all diagnoses
  - ◻ Sepsis evaluation
  - ◻ HIE (Hypoxic ischemic encephalopathy, birth asphyxia; 5-minute Apgar score < 6; initial pH < 7.0)
  - ◻ Seizures (can be clinical or electrographic or both)
  - ◻ Hypoglycemia (blood glucose < 35)
  - ◻ Hyperbilirubinemia (including need for phototherapy or exchange transfusion)
  - ◻ Dehydration
  - ◻ Metabolic evaluation (inborn error of metabolism, etc.)
  - ◻ Chromosomal anomaly (indicate if trisomy 21 or other chromosomal anomaly, unspecified)
  - ◻ Congenital heart disease
  - ◻ NEC
  - ◻ Omphalocele, Gastroschisis
  - ◻ Other surgical evaluation
  - ◻ Meningomyelocele
  - ◻ Other intracranial abnormalities
  - ◻ SGA (< 3%ile)
  - ◻ Other /specify____

- Length of time baby was in the NICU on the day of admission hours ___________
  mins ___________

Initials of person completing this form _____________
Date completed ____________________

Site subject number ______
Database subject number ______

DISCHARGE FORM

<u>Disposition/"Status"</u>

- ◻ Discharged home prior to120 days of age
- ◻ Still in NICU at $\geq$ 120 days of age
- ◻ Transferred to community hospital, other facility, or other hospital unit for convalescent care prior to 120 days
- ◻ Transferred to another hospital, facility or hospital unit, for escalation of care prior to 120 days
- ◻ Died in hospital at ≤ 120 days

<u>Date</u> of disposition/"status" __/__/____

<u>Measurements</u> at "status"

- Weight in grams __________
- Length in cms __________
- Head circumference in cms __________

<u>Discharge medications</u>

- ◻ antibiotics for urinary tract infection (UTI) prophylaxis
  - ◻ yes
  - ◻ no
- ◻ diuretics
  - ◻ yes
  - ◻ no
- ◻ antihypertensives:
  - ◻ yes
  - ◻ no

Initials of person completing the form _________________________
Date completed _____________________________

Site subject number __________
Database subject number ______

PROLONGED LENGTH OF STAY (>120 DAYS) FORM

Disposition/"Status"

- □ Discharged home prior to one year of age
- □ Still in NICU at > 1 year of age
- □ Transferred to another facility
- □ Died in hospital at > 120 days

Date of disposition/"status" __/__/____

Measurements at "status"

- □ Weight in grams _________
- □ Length in cms _________
- □ Head circumference in cms _________

Discharge medications

- □ antibiotics for urinary tract infection (UTI) prophylaxis
  - □ yes
  - □ no
- □ diuretics
  - □ yes
  - □ no
- □ antihypertensives:
  - □ yes
  - □ no

Reason for NICU stay > 120 days (check all that apply)

- □ Pulmonary
- □ Cardiac
- □ Neurologic
- □ GI
- □ Multiple malformations
- □ Sepsis/infection
- □ Renal
  - o With RRT
  - o Without RRT
- □ Other _____________________________

Last creatinine obtained between 120 days and "status" _____________mg/dL

Site subject number ______
Database subject number ______

Discharge Diagnoses

- □ Cardiac
  - □ PDA confirmed
    - □ Self-resolved
    - □ treated with indomethacin only
    - □ surgical ligation with prior indomethacin treatment
    - □ surgical ligation without prior indomethacin treatment
  - □ Anatomic cardiac anomaly
  - □ Systemic hypertension
    - □ no medications at discharge
    - □ medications at discharge

- □ Pulmonary
  - □ BPD
    - □ requiring continued mechanical ventilation at 36 weeks' CGA
    - □ requiring continued non-invasive ventilation or CPAP at 36 weeks' CGA
    - □ requiring supplemental oxygen by nasal cannula or hood at 36 weeks' CGA
  - □ Persistent pulmonary hypertension confirmed
    - □ not requiring iNO or ECMO
    - □ requiring iNO
    - □ requiring ECMO

- □ Neurologic
  - □ IVH or PVL
    - □ None
    - □ IVH grade I

Initials of person completing the form ________________________
Date completed ____________________________

- ◻ IVH grade II
- ◻ IVH grade III
- ◻ IVH grade IV
- ◻ PVL (can be alone or in conjunction with IVH)
- ◻ HIE (hypoxic ischemic encephalopathy/birth asphyxia)
- ◻ Seizures

- ◻ GI
  - ◻ NEC
    - ◻ Bell Stage 2 – medically treated
    - ◻ Bell Stage 2 – surgically treated
  - ◻ Jaundice requiring an exchange transfusion

- ◻ Hematologic
  - ◻ DIC (disseminated intravascular coagulation)

- ◻ Infectious Disease
  - ◻ Culture negative sepsis (negative culture but treated with antibiotics for ≥ 5d)
  - ◻ Bacteremia
  - ◻ Viremia
  - ◻ Meningitis/encephalitis, include both bacterial and viral infections
- ◻ Metabolic abnormalities
- ◻ Endocrine abnormalities
- ◻ Genetic abnormalities
- ◻ Other major diagnoses
  - ◻ Specify___________________________________

Initials of person completing the form ________________________
Date completed ____________________________

Site subject number ______
Database subject number ______

Renal diagnoses

- □ Nephrology consult obtained during this admission.
- □ Acute kidney injury (coded) or acute renal failure coded. Includes ICD-9 codes 584.*
- □ Urinary tract infections. Please include only if there was a positive urine culture
- □ Medullary nephrocalcinosis/calcifications/kidney stones. Must be documented on renal ultrasound.

Congenital abnormalities of the kidney. (use most severe on Renal US or Discharge summary) Please circle all that apply from the list provided. See MOP for description

	Right	Left
Hypoplasia/Dysplasia	Yes NO	Yes NO
Multicystic Dysplastic kidney	Yes NO	Yes NO
Renal agenesis	Yes NO	Yes NO
Polycystic kidney disease		
	Yes, recessive	
	Yes, dominant	
	Unknown	
Horseshoe kidney	YES<br>NO	
Renal Ectopia	Yes NO	Yes NO
Hydronephrosis	Yes NO<br>Mild MOD Severe	Yes NO<br>Mild MOD Severe
UPJ	Yes NO	Yes NO
Hydroureter	Yes NO	Yes NO
Duplicated System	Yes NO	Yes NO
Posterior urethral valves	YES<br>NO	
Vesicoureteral reflux	Yes NO	Yes NO
	1 2 3 4 5	1 2 3 4 5
	Abnormality Present	
Urethral stricture	YES<br>NO	
Bladder exstrophy	YES<br>NO	
Neurogenic Bladder	YES<br>NO	
Prune Belly Syndrome	YES<br>NO	

Initials of person completing the form ________________________
Date completed ____________________________

Site subject number ______
Database subject number ______

Renal replacement therapy: *This section should be completed by the nephrologist member of your group.*

- ○ yes
- ○ no

- If YES,

  How many days did the patient receive any form of renal replacement therapy during the hospitalization? _______________

  ○ Modality (please choose all that apply):

  - □ Peritoneal dialysis
  - □ intermittent hemodialysis
  - □ CRRT
  - □ CRRT with ECMO
  - □ SLED

  If CRRT (or CRRT with ECMO), Indicate modality

  - □ *CVVH*
  - □ *CVVHD*
  - □ *CVVHDF*
  - □ *SCUF*

  If CRRT Indicate machine (please choose all that apply)

  - □ *Prismaflex*
  - □ *NxStage*
  - □ *Aquadex*
  - □ *Fresenius*
  - □ *In-line filter (for CRRT/ECMO only)*

  Type of anticoagulation (Only for ECMO with CRRT and CRRT alone)

  - □ Heparin
  - □ Citrate/calcium,
  - □ None
  - □ Unknown

Initials of person completing the form ________________________
Date completed ____________________________

Site subject number _____
Database subject number _____

SCREENING AND INTAKE FORM

Demographics

1. Gender __________
    a. male,
    b. female,
    c. ambiguous
2. Ethnicity ___________
    a. Hispanic/Latino/Spanish origin;
    b. Non-Hispanic/non-Latino/non-Spanish origin;
    c. unknown
3. Race - choose all that apply ___________
    a. White/Caucasian
    b. Black/African-American
    c. American Indian/Alaska native;
    d. Asian;
    e. Native Hawaiian or other Pacific Islander;
    f. unknown
    g. other
4. Date of birth ___________
5. Date of NICU admission ___________

Inclusion/Exclusion Criteria

Eligibility – must indicate yes to both 1 and 2.

- Inclusion criterion:
    1. All infants admitted to a participating center NICU (level 2 or 3) who do not meet exclusionary criteria

       Yes □
       No □

    2. Infants who receive at least 48 hours of intravenous hydration. This includes iv fluids to provide hydration and/or nutrition and does not include iv fluids solely for administration of medications.

       Yes □
       No □

- Exclusion criteria – <u>any</u> <u>one</u> will exclude patient
    1. Admission to the NICU at ≥ 14 days of age

       Yes □
       No □

Initials of the person completing this form __________________
Date completed ____

Site subject number _____
Database subject number _____

2. Congenital heart disease requiring surgery within the first 7 days

Yes □
No □

3. Lethal chromosomal anomalies

Yes □
No □

4. Infants who die at < 48 hours of age

Yes □
No □

Initials of the person completing this form ___________________
Date completed ____

Site subject number ______
Database subject number ______

WEEK ONE DATA

PLEASE NOTE: DAY OF BIRTH = DAY 1

Physiologic parameters: Please enter the highest, lowest and value closest to midnight (first)

	Day 1	Day 2	Day 3	Day 4	Day 5	Day 6	Day 7
date							
Weight (g)							
Systolic BP							
Highest							
Lowest							
First							
Diastolic BP							
Highest							
Lowest							
First							
Mean Arterial BP							
Highest							
Lowest							
First							

Initials of person completing the form
Date completed

Site subject number ______
Database subject number ______

Respiratory parameters:

1 ECMO

2 High frequency ventilation

3 Conventional ventilation

4 Noninvasive ventilation

5 CPAP

6 Nasal cannula

7 Oxyhood

8 No respiratory support

	Day 1	Day 2	Day 3	Day 4	Day 5	Day 6	Day 7
date							
Mode							
Max mean airway pressure							
Max FiO2							

Initials of person completing the form
Date completed

Site subject number _______
Database subject number _______

Fluid balance:

Fluid IN

Day	date	Quantifiable IV fluids No=0 Yes=1	If yes, IV fluid volume	Quantifiable enteral fluids No=0 Yes=1	If yes, Enteral fluid volume
1					
2					
3					
4					
5					
6					
7					

Fluid Out

Day	date	Was there fluid out in 24 hour period? No = 0 Yes = 1	Quantifiable? No=0 Yes=1	If yes, Total volume
1				
2				
3				
4				
5				
6				
7				

Urine output

Day	date	Was there urine out in 24 hour period? No = 0 Yes = 1	Quantifiable? No=0 Yes=1	If yes, Total volume
1				
2				
3				
4				
5				
6				
7				

Initials of person completing the form
Date completed

Site subject number ______
Database subject number ______

Medications: "0" = no; "1" = yes for any part of that day

	Day 1	Day 2	Day 3	Day 4	Day 5	Day 6	Day 7
date							
Aminoglycoside							
Vancomycin							
Piperacillin-Tazobactam							
Amphotericin							
Acyclovir							
Indomethacin							
ibuprofen							
hydralazine							
ACE-inhibitors							
Dopamine							
Dobutamine							
Milrinone							
Epinephrine							
Norepinephrine							
Furosemide							
Bumetanide							
Chlorothiazide							
Ethacrynic acid							
Spironolactone							
Theophylline							
Caffeine							

Initials of person completing the form
Date completed

Site subject number ______
Database subject number ______

Laboratory values: Include "worst" for day if more than one value obtained (highest creatinine, highest BUN, lowest albumin, lowest hemoglobin or hematocrit, highest and lowest sodium)

	Day 1	Day 2	Day 3	Day 4	Day 5	Day 6	Day 7
date							
BUN							
Albumin							
Hemoglobin							
Hematocrit							
Sodium							
Highest							
Lowest							
Blood culture<br>0 = negative<br>1 = positive<br>2 = not done							
CSF culture<br>0 = negative<br>1 = positive<br>2 = not done							
Urine culture<br>0 = negative<br>1 = positive<br>2 = not done							

Initials of person completing the form
Date completed

Site subject number ________
Database subject number ________

WEEKLY DATA

Physiologic parameters: Please enter the highest, lowest and value closest to midnight for **the day closest to the first day of each week (day 8, 15, 22, 29, etc.)**

	Week 2	Week 3	Week 4	Week 5	Week 6	Week 7	Week 8
date							
Weight (g)							
Systolic BP							
Highest							
Lowest							
First							
Diastolic BP							
Highest							
Lowest							
First							
Mean arterial pressure							
Highest							
Lowest							
First							
	Week 9	Week 10	Week 11	Week 12	Week 13	Week 14	Week 15
date							
Weight (g)							
Systolic BP							
Highest							
Lowest							
First							
Diastolic BP							
Highest							
Lowest							
First							
Mean arterial pressure							
Highest							
Lowest							
First							
	Week 16	Week 17	Week 18				
date							
Weight (g)							
Systolic BP							
Highest							
Lowest							
First							
Diastolic BP							
Highest							

Initials of person completing form ________________
Date completed _________________

Site subject number ________
Database subject number ________

Lowest							
First							
Mean arterial pressure							
Highest							
Lowest							
First							

Initials of person completing form _________________
Date completed __________________

Site subject number ________
Database subject number ________

Respiratory parameters:

1 ECMO
2 High frequency ventilation
3 Conventional ventilation
4 Noninvasive ventilation
5 CPAP
6 Nasal cannula
7 Oxyhood
8 No respiratory support

	Week 2	Week 3	Week 4	Week 5	Week 6	Week 7	Week 8
date							
Mode							
Max mean airway pressure							
Max FiO2							
	Week 9	Week 10	Week 11	Week 12	Week 13	Week 14	Week 15
date							
Mode							
Max mean airway pressure							
Max FiO2							
	Week 16	Week 17	Week 18				
date							
Mode							
Max mean airway pressure							
Max FiO2							

Initials of person completing form ________________
Date completed _________________

Site subject number ________
Database subject number ________

Fluid balance: Enter intake/output **for first day of each week (day 8, 15, 22, 29, etc)**

Intake

Week	Date	Quantifiable IV fluids? No=0 Yes=1	Total IV fluids volume	Quantifiable Enteral fluids? No=0 Yes=1	Total enteral fluid volume
2					
3					
4					
5					
6					
7					
8					
9					
10					
11					
12					
13					
14					
15					
16					
17					
18					

Total fluid output (urine plus other)

Week	Date	Was there fluid output? No = 0 Yes = 1	Quantifiable? No=0 Yes=1	Total volume
2				
3				
4				
5				
6				
7				
8				
9				
10				
11				
12				
13				
14				
15				
16				
17				

Initials of person completing form ________________
Date completed _________________

Site subject number ________
Database subject number ________

18				

Initials of person completing form ________________
Date completed _________________

Site subject number ________
Database subject number ________

<u>Medications</u>: "0" = no; "1" = yes **for the first day of each week**

	Week 2	Week 3	Week 4	Week 5	Week 6	Week 7
date						
Aminoglycoside						
Vancomycin						
Piperacillin-Tazobactam						
Amphotericin						
Acyclovir						
Indomethacin						
ibuprofen						
hydralazine						
ACE-inhibitors						
Dopamine						
Dobutamine						
Milrinone						
Epinephrine						
Norepinephrine						
Furosemide						
Bumetanide						
Chlorothiazide						
Ethacrynic acid						
Spironolactone						
Theophylline						
Caffeine						
	Week 8	Week 9	Week 10	Week 11	Week 12	Week 13
date						
Aminoglycoside						
Vancomycin						
Piperacillin-Tazobactam						
Amphotericin						
Acyclovir						
Indomethacin						
Ibuprofen						
hydralazine						
ACE-inhibitors						
Dopamine						
Dobutamine						
Milrinone						

Initials of person completing form ________________
Date completed _________________

Site subject number ________
Database subject number ________

Epinephrine						
Norepinephrine						
Furosemide						
Bumetanide						
Chlorothiazide						
Ethacrynic acid						
Spironolactone						
Theophylline						
Caffeine						
	Week 14	Week 15	Week 16	Week 17	Week 18	
date						
Aminoglycoside						
Vancomycin						
Piperacillin-Tazobactam						
Amphotericin						
Acyclovir						
Indomethacin						
Ibuprofen						
hydralazine						
ACE-inhibitors						
Dopamine						
Dobutamine						
Milrinone						
Epinephrine						
Norepinephrine						
Furosemide						
Bumetanide						
Chlorothiazide						
Ethacrynic acid						
Spironolactone						
Theophylline						
Caffeine						

Initials of person completing form _________________
Date completed __________________

Site subject number ________
Database subject number ________

Laboratory values: Include "worst" **for first day of each week** if more than one value obtained (highest creatinine, highest BUN, lowest albumin, lowest hemoglobin/hematocrit, highest and lowest sodium)

	Week 2	Week 3	Week 4	Week 5	Week 6	Week 7
Date						
BUN						
Albumin						
Hemoglobin						
Hematocrit						
Sodium						
Highest						
Lowest						
Blood culture<br>0 = negative<br>1 = positive<br>2 = not done						
CSF culture<br>0 = negative<br>1 = positive<br>2 = not done						
Urine culture<br>0 = negative<br>1 = positive<br>2 = not done						

	Week 8	Week 9	Week 10	Week 11	Week 12	Week 13
Date						
BUN						
Albumin						
Hemoglobin or hematocrit						
Sodium						
Highest						
Lowest						
Blood culture<br>0 = negative<br>1 = positive<br>2 = not done						
CSF culture<br>0 = negative<br>1 = positive<br>2 = not done						
Urine culture<br>0 = negative<br>1 = positive						

Initials of person completing form ________________
Date completed _________________

Site subject number ________
Database subject number ________

2 = not done						
	Week14	Week 15	Week 16	Week 17	Week 18	
Date						
BUN						
Albumin						
Hemoglobin or hematocrit						
Sodium						
Highest						
Lowest						
Blood culture<br>0 = negative<br>1 = positive<br>2 = not done						
CSF culture<br>0 = negative<br>1 = positive<br>2 = not done						
Urine culture<br>0 = negative<br>1 = positive<br>2 = not done						

Initials of person completing form _________________
Date completed __________________

Site subject number ________
Database subject number ________

Creatinine Values

Date	Time (if more than one creatinine level on the same date)	Value

Initials of person completing form _________________
Date completed __________________

Site subject number ________
Database subject number ________

Initials of person completing form ________________
Date completed _________________
