# Supplementary material for: Assessment of Worldwide Acute Kidney Injury Epidemiology in Neonates: Design of a Retrospective Cohort Study
Source: Front Pediatr. 2016 Jul 19;4:68. doi: 10.3389/fped.2016.00068 (PMC4950470; doi:10.3389/fped.2016.00068)
Supplement: Supplementary file 2 [file Data_Sheet2.PDF]

General Information:

This is a retrospective study that is not intended to be comprehensive. We are targeting information that is most likely to be related to renal function in neonates.

We are requesting more detailed information from the first 7 days after birth and then collecting "snapshot" data weekly thereafter. Data need only be extracted from the infant's chart. If maternal information or initial resuscitation and hospitalization information for outborn babies is not recorded in the infant's chart, you may indicate "unknown". However, if you have ready access to mother's chart, all available information should be entered.

If using the paper forms for initial data extraction, please initial and date all CRFs and maintain these forms according to good clinical practice guidelines. The forms closely mirror the electronic forms though the eCRFs will have "triggers" designed to eliminate the need to respond to questions that are not relevant for that baby. Required responses will lead to queries if no data are entered or if the data fall outside the established ranges. Please respond to these queries promptly.

Please direct all questions to Lynn Dill. She will triage these questions; you will get a response ASAP. Review the weekly FAQ email as others in the collaborative may have already asked your question.

FINAL

SCREENING AND INTAKE FORM

Basic demographic information should be entered for all NICU admissions (level 2 and 3 only, not babies who go to the normal newborn nursery) within the study period. This will allow complete ascertainment of the NICU population from which the study sample was drawn. Based on institutional IRB guidelines, name and/or medical record number can be collected but not entered into the study database. This PHI should be used only to make sure all patients are accounted for and to assist in accurate collection of data, especially if all data cannot be collected at one time.

Demographics

1. Gender – enter male, female, ambiguous
2. Ethnicity – enter Hispanic/Latino/Spanish origin; Non-Hispanic/non-Latino/non-Spanish origin; other; or unknown as documented in the medical record. If mother's but not baby's ethnicity is available, enter mother's information.
3. Race – enter American Indian/Alaska native; Asian; Black or African American; Native Hawaiian or other Pacific Islander; White; other; or unknown as documented in the medical record. If mother's but not baby's race is available, enter mother's information.
4. Date of birth– this information is needed as an "anchor" for subsequent calculations and determinations of timing of data collection. Your IRB submission should specify a "limited data set" with inclusion of the date of birth. Enter as DD/MM/YYYY
5. Date of NICU admission – enter as DD/MM/YYYY

FINAL

Inclusion/Exclusion Criteria:

Eligibility – must meet both inclusion criteria and none of the exclusion criteria

- Inclusion criterion: YES to BOTH
    1. All infants admitted to a participating center NICU (level 2 or 3) who do not meet exclusionary criteria
    2. Infants who receive at least 48 hours of intravenous hydration/nutrition. This includes iv fluids to provide hydration and/or nutrition and does not include iv fluids solely for administration of medications.

- Exclusion criteria: Any ONE will exclude the baby from the study
    1. Admission to the NICU at ≥ 14 days of age
    2. Congenital heart disease requiring surgery within the first 7 days

       Infants with VSD, ASD, PDA are NOT excluded unless they are transferred out of the NICU in < 7 days for surgical repair. Premature babies who remain in the NICU for more than 7 days to gain weight and grow in preparation for surgery are NOT excluded. Infants cared for in the NICU for less than 7 days prior to surgery and/or transferred to the PICU are excluded.

    3. Lethal chromosomal anomalies or conditions; any baby not expected to survive to discharge

       Including Trisomy 13, 18, anencephaly

    4. Infants who die at < 48 hours of age

FINAL

BASELINE FORM

Maternal information:

- Enter maternal age at delivery in years
- Enter gravida (number of pregnancies), parity (number of living children), including the current pregnancy; if multiple gestation include all under parity. Use the "G" and "P" information documented in the chart whenever possible (i.e., you do not have to calculate these numbers yourself).
- Indicate if there is evidence of maternal infections at or near the time of delivery including bacterial and viral infections.
  - Mark as intrapartum bacterial infection if mother treated for chorioamnionitis
  - Do not mark as intrapartum bacterial infection if mother treated only with penicillin for GBS prophylaxis or with latency antibiotics such as azithromycin
  - Mark as intrapartum viral infection if mother HIV positive or if she was diagnosed with Herpes infection at or near the time of delivery
  - Do not mark as intrapartum viral infection if only on Valtrex for prophylaxis
- Enter diabetes for any category of diabetes (juvenile/prepregnancy or gestational) and regardless of mode of management (diet controlled, oral hypoglycemics, or insulin)
- Enter hypothyroidism if documented in infant or maternal medical record or transfer information as present during this pregnancy, with or without current medications
- Enter chronic hypertension if noted prior to this pregnancy, with or without current medications
- Indicate if history of kidney disease in mother including congenital kidney abnormalities (solitary kidney, dysplastic kidney, etc.), acquired disease (diabetic nephropathy, nephrotic syndrome, lupus nephritis, etc.), kidney transplantation, regardless of current treatment (medication or dialysis); exclude frequent UTI without confirmed anatomic abnormality; exclude maternal kidney stones.
- Enter pre-eclampsia if documented in infant or maternal record or transfer information, with or without current medications; also include any mother with pregnancy –induced hypertension in this category.
- Enter eclampsia if seizures associated with maternal hypertension or HELPP syndrome
- Enter IUGR <u>if noted and documented during pregnancy</u>; small for gestational age will be ascertained based on dating and birthweight so no need to calculate
- Enter oligohydramnios if documented in infant or maternal record or transfer information
- Enter polyhydramnios if documented in maternal record or transfer information
- Enter maternal hemorrhage if documented in infant or maternal record or transfer information; do not include "bloody show." Answer "yes" if the hospital notes indicate a clinically significant amount of bleeding; there are no other qualifying criteria needed for this (e.g., need for transfusion).
- For multiple gestation, indicate if all fetuses survived to birth or if there was an antenatal demise of one or more. Enter "unknown" if a transfer and no information is available in transfer information about other fetuses
- If multiple gestation, indicate if twin-twin transfusion was documented; if not part of a multiple gestation, enter "no" or do not check on the paper form

FINAL

- Enter by category all drugs documented as having been used during this pregnancy including maternal steroids for fetal maturation (betamethasone); ACE-inhibitors (captopril, enalapril, lisinopril, benazepril, fosinopril, quinapril, enalaprilat), NSAIDs (including aspirin, ibuprofen, paracetamol, other over-the-counter pain relievers), beta blockers (propranolol, atenolol, carvedilol, metoprolol, esmolol, labetalol), calcium channel blockers (amlodipine, felodipine, isradipine, nicardipine), vasodilators (hydralazine, minoxidil, nitroprusside), central alpha-agonists (clonidine), indomethacin/Indocin (when given intrapartum for tocolysis – often prescribed in preterm labor or preterm rupture of membranes to help delay delivery and facilitate treatment with antenatal steroids), illicit drugs (such as cocaine, heroin, THC, and any other street drugs; also include drugs used in drug treatment programs such as methadone, subutex) by history or drug screening, tobacco, alcohol, and SSRIs (citalopram/Celexa®, escitalopram/Lexapro®, fluvoxamine/Luvox®, paroxetine/Paxil®, fluoxetine/Prozac, ® sertraline/Zoloft®); heparin or warfarin. Enter "unknown" if information is unclear or if maternal drug history was not taken.
- Indicate if known assisted conception (intrauterine insemination, hormonally induced ovulation, gamete transfer "GIFT")
- Intrapartum complications: Indicate if documentation of nuchal cord, meconium staining or in amniotic fluid, severe maternal vaginal bleeding, cord rupture, shoulder dystocia; choose all that apply. Enter none if no intrapartum complications are noted in the record. Enter unknown if there is no information available.

FINAL

Neonatal information

- Site of delivery - indicate if born at study institution (“inborn” including delivery room, birth center, or emergency department) or born elsewhere (“outborn” including transfer from another institution, born at home, born en route to hospital)
- Gestational age at birth, enter as weeks and days; if no days are documented enter “0” days
    - Record the best estimate in weeks and days using the following hierarchy
        - Best OB estimate: last menstrual period, OB parameters, and/or early prenatal ultrasound
        - Best neonatologist estimate: Ballard or Dubowitz criteria
    - If estimated date of confinement (expected due date) or other details on gestational age are not available and baby only identified as “full term”, enter “40” weeks, “0” days
- Birthweight, enter as grams; if no birthweight available from birth, enter weight on admission
- Length at birth (or if not available, at admission), enter as centimeters – round to nearest whole number
- Head circumference at birth (or if not available, at admission), enter as centimeters – round to nearest whole number
- Admission temperature (°C): Enter temperature at admission to nursery at the hospital of birth. If born outside an institution, enter first recorded temperature. If no temperature available at place of birth, enter “unknown.” Do not include an admission temperature at a transferring institution if the baby is more than 12 hours old. This will be used to calculate a severity of illness score – the scoring system requires a temperature within the first 12 hours of life.

- Mode of delivery, please specify from list provided

Resuscitation

- Apgar scores, enter all that are documented. Leave blank if none documented for that time interval.
- Cord blood gas results: enter all that are available for both arterial and venous; enter base excess as a positive number (if > 0 enter as “0”). If a cord gas is obtained, but the vessel is not specified enter as such (“vessel unspecified”).
- If no cord blood gases are available, enter a blood gas obtained during the first hour, if available. Enter the pH and base excess from any source (arterial, venous, capillary). Enter base excess as a positive number (if > 0 enter as “0”)
- Enter all resuscitative measures provided to the infant in the delivery room (list provided):
    - None (aside from routine drying and stimulation)
    - supplemental oxygen (any oxygen > 21% provided by any means including oxyhood, “free flow”, face mask, CPAP, intubation);
    - PPV (positive pressure ventilation with face mask and bag – self inflating or anesthesia, or NeoPuff);
    - intubation (for any duration of positive pressure ventilation; do not include if solely for suctioning of meconium or blood from the airway);
    - chest compressions;
    - epinephrine (either intratracheal or intravenous);

FINAL

- normal saline (fluid bolus for poor perfusion, low blood pressure);
- blood transfusion (whole blood or red blood cells).
- “unknown” if no information is available.

<u>Reason for admission</u>

- Enter the reason(s) for admission from the list provided.- choose all that apply; information provided by review of admission note/admission diagnosis or problem list
    - “prematurity” if gestational age at birth is < 35 weeks;
    - Respiratory symptoms (requiring observation and close monitoring and support no greater than supplemental oxygen via oxyhood or low flow nasal cannula, < 2L/min)
    - Respiratory failure (continued need for respiratory support including conventional ventilation, high frequency ventilation, non-invasive ventilation, CPAP, or High Flow Nasal Cannula ≥ 2L/min)
    - Sepsis evaluation
    - HIE (Hypoxic ischemic encephalopathy, birth asphyxia; 5-minute Apgar score < 6; initial pH < 7.0)
    - Seizures (can be clinical or electrographic or both)
    - Hypoglycemia (blood glucose < 35)
    - Hyperbilirubinemia (including need for phototherapy or exchange transfusion)
    - Dehydration
    - Metabolic evaluation (inborn error of metabolism, unexplained lactic acidosis, hyperammonemia, etc.)
    - Chromosomal anomaly (indicate if trisomy 21 or other chromosomal anomaly, unspecified)
    - Congenital heart disease (not surgically corrected or palliated in the first 7 days)
    - NEC (necrotizing enterocolitis)
    - Omphalocele, Gastroschisis
    - Other surgical evaluation (including but not limited to NEC, TE fistula, congenital diaphragmatic hernia, intestinal atresias, etc.)
    - Meningomyelocele
    - Other intracranial abnormalities (anencephaly, brain malformations, hydrocephaly, etc.)
    - SGA (< 3%ile)
    - Other (specify_____________________________________)

- Length of time baby was in the NICU on the day of admission – please enter the number of hours and the number of minutes in the appropriate fields. Example: if baby was admitted at 10:35pm, please enter “1” under hours and “25” under minutes. If the baby was admitted after 11pm, enter “0” for hours. This information will be used to calculate urine output in “ml/kg/hr.”

FINAL

WEEK ONE DATA FORM

Data is limited to the first week after birth. If a baby is admitted after the day of birth, start data entry on the appropriate day of life (e.g., the day of life on the day of admission) and leave the initial days blank. Babies will have data for 1 – 7 days, depending on age at admission to the NICU. The electronic database will ask at the beginning which days the baby was in the NICU then populate data fields to be completed only for those dates.

- For all data forms, **Day 1 = day of birth**. Use "calendar day", not first 24 hours after birth. Thus, day 1/day of admission may vary from < 1 hour to 24 hours; all other days will encompass a 24 hour period.
  (The number of hours in the NICU on the day of admission will be indicated on the Baseline form.)

- Weight: Please enter the weight in gram taken closest to but after midnight for that calendar day.

- Blood pressure: Please enter the value closest to but after midnight on that calendar day ("first") and the highest and lowest value for the day. If only one value is available for that day, please enter that value for all fields (first, highest and lowest).

- Respiratory parameters: Please enter values for highest level of support for that day; enter highest supplemental oxygen for highest level of support. For example, if on mechanical ventilation at a maximum of 30% FiO2 and on nasal cannula at 50% during that day, enter mechanical ventilation and 30% O2. If on ECMO, enter circuit FiO2 and leave mean airway pressure blank. If on nasal cannula or oxyhood, enter FiO2; you will not be prompted to include a mean airway pressure.

- Fluid balance:
  - The database will have prompts regarding whether fluid input/output was 1) present and 2) quantifiable. This will allow us to distinguish babies with oliguria/anuria from those with urine output that was counted only in terms of number of diapers, e.g.,
  - For Total fluid IN, include maintenance fluids, blood product transfusions, and fluid boluses. If at your institution your eMR includes the fluid administered as part of medication infusion/flushes as part of total fluids, include this volume – there is no need to subtract that volume. If at your institution your eMR does not include fluid administered as part of medication infusions/flushes, no need to include this volume.
  - Please indicate whether the fluid intake is quantifiable (e.g., listed in ml as opposed to number of breast feeds or "x 1.") Enter quantifiable IV fluid and enteral fluids in ml separately as prompted by database or in the appropriate column on paper forms.
  - For Total fluid OUT, include total urine output as well as other losses such as chest tube drainage, ostomy drainage, G-tube drainage, etc. for each 24 hour period
  - For Urine output, please enter for each 24 hour period. Please indicate if urine output was able to be quantified (e.g., documented in ml as opposed to number of diapers or "x 1.") Enter quantifiable output in ml for each 24 hour period as prompted by the data base.

- If it is clearly documented that there was no urine output (e.g., “0” is documented each shift), then enter “0” under ml.
- If either fluid intake or output was not collected, please indicate this at the appropriate prompt (“Not done/not available/unknown”).
- Please calculate IN and OUT based on 0001 – 2400. If your electronic medical record is set up to automatically calculate 24 hour intake and output 2301 – 2300 or 0100 - 0059, you may use those numbers. Any other automatically calculated 24 hour interval (eg., 0700 – 0659) is NOT acceptable and total intake and output will have to be calculated “by hand”.

- Medications: Indicate the administration of medications on the list. Check the box for any medication the patient received the medication at any point during that calendar day (i.e., patient does not need to have received the medication for the whole day). Please confirm that the medication was administered, not just ordered. Please choose all that apply. This is not meant to be an exhaustive list of all medications administered in the NICU. We are interested primarily in drugs that may affect renal function.
  - Aminoglycosides include: gentamicin, amikacin, tobramycin, netilimicin

- Laboratory values: Include “worst” for day if more than one value obtained (highest BUN, lowest albumin, lowest hemoglobin or hematocrit, highest/lowest sodium).
  - For creatinine, include ALL creatinine values obtained. A separate table is provided for this purpose to encompass the entire hospital stay
  - Appropriate conversions will be performed “behind the scenes” based on the units of measurement at different institutions. Please indicate the units used at your institution. You may enter either hematocrit or hemoglobin; no need to enter both.

FINAL

WEEKLY DATA FORMS:

- For all data forms, Day 1 = calendar day of birth; Week 2 = days of life 8-14; Week 3 = days of life 15 – 21; Week 4 = days of life 22 – 28; etc. The dates will populate automatically in the database based on the date of birth
- Weight: Please enter the weight in gram taken closest to but after midnight for first day of the week. If no weight is available on the first day, then enter the first weight available for that week.
- Blood pressure: Please enter the highest and lowest (best and worst) values for each parameter for the first day of each week (i.e., days 8, 15, 22, etc.). If no values are available on the first day of the week, then enter the first value available for that week. If there is only one measure (e.g., only one blood pressure for that week), enter that same value in all fields (highest, lowest and first).
- Respiratory parameters: Please enter the highest level of support for the first day of each week; use the highest level of support for that day's data (i.e., if on both mechanical ventilation and nasal cannula, count that day as mechanical ventilation); enter highest supplemental oxygen for highest level of support for that week (i.e., if on mechanical ventilation at 30% max and on nasal cannula at 50% max during that week, enter 30% O2). If on ECMO, enter circuit FiO2 and leave mean airway pressure blank. If on nasal cannula or oxyhood, please enter the FiO2 and leave mean airway pressure blank.
- Fluid balance:
    - Enter the total fluid intake for the first day of the week; include parenteral fluids, enteral fluids, blood product transfusions and fluid boluses if quantified in ml. Enter IV and enteral fluids separately as for the daily form.
    - Enter the total fluid output for the first day of the week; include urinary output, chest tube drainage, ostomy drainage, G-tube drainage if quantified in ml. Enter as for daily form.
- Medications: Indicate if the baby received the listed medication on the first day of each week. This is only a "snap shot" so there is no need to indicate what medications babies receive during other days of the week.
- Laboratory values: Enter values for the first day of the week. If no value is available for the first day of the week, then enter the first value available for that week. Include "worst" for that day if more than one value obtained (highest BUN lowest albumin, lowest hemoglobin or hematocrit, highest sodium). You may enter either hematocrit or hemoglobin, whichever is available for that week, if any. Indicate if positive culture obtained during that week (use first value available), no need to specify organism. For creatinine, include ALL creatinine values obtained. A separate table is provided for this purpose to encompass the entire hospital stay. If more than one creatinine is obtained on a given day, indicate the times they were drawn. You need not enter time if only one creatinine on a calendar day.

FINAL

DISCHARGE FORM

Disposition/"Status": Please choose from drop-down menu

- Discharged home prior to 120 days of age
- Still in NICU at $\geq$ 120 days of age
- Transferred to community hospital, other facility, or other hospital unit for convalescent care prior to 120 days of age
- Transferred to another hospital, facility or hospital unit, for escalation of care prior to 120 days of age
- Died in hospital prior to 120 days of age
    a. If died, please remember to complete Discharge Diagnoses; discharge medications will not apply; measurements at "status" should be entered if available.

Date of disposition/"status", enter as DD/MM/YYYY

- Measurements at "status" – enter data recorded at discharge from NICU (home or other unit/facility) or 120 days of age, whichever is earlier. If measurements not available at "status" date, enter the information from the day closest to and before (not after) "status" date.
- Weight in grams
- Length in cms
- Head circumference in cms

Discharge medications – you need only indicate if the baby was sent home on the listed categories of medications.

- antibiotics for urinary tract infection (UTI) prophylaxis in patients with urinary tract abnormalities including but not limited to VUR, hydronephrosis, or neurogenic bladder requiring intermittent catheterization: check "yes" if discharged on amoxicillin, trimethoprim/sulfa, nitrofurantoin, other antibiotic with clear indication as being for UTI prophylaxis, none
- diuretics: indicate if discharged on diuretics for any reason including congenital heart disease, BPD or chronic kidney disease. Check "yes" if discharged on furosemide, spironolactone, hydrochlorothiazide, other, or any combination
- Antihypertensives: check "yes" if discharged on captopril, enalapril, isradipine, amlodipine, propranolol or other antihypertensive medication with clear indication as being for blood pressure control.

Discharge Diagnoses: include all from drop down menu if confirmed during this hospitalization whether or not resolved at time of "status" for each category listed (cardiac, pulmonary, neurologic, GI, hematologic, infectious disease, genetic, endocrine, metabolic). Include only diagnoses made between admission and "status". This list is not exhaustive, but highlights those diagnoses that may be most related to the development of renal injury in neonates. For example, we have included under Pulmonary only BPD and PPHN and no other pulmonary diagnoses such as Transient Tachypnea of the Newborn (TTN), Respiratory Distress Syndrome (RDS), or Congenital Cystic Adenomatous Malformation (CCAM). We did not include any specific diagnoses under Genetic, Metabolic, or Endocrine. Diagnoses

such as Aneuploidy, Inborn Errors of Metabolism, or Congenital Adrenal Hyperplasia (that may be diagnosed after admission and therefore not appear on the admission diagnosis list). Simply indicate "yes" in the appropriate category.

If your baby has a major medical diagnosis not elsewhere captured on this form and you think that it should be reflected in the data, please check "yes" for "**Other major diagnoses**" and type in the diagnosis in the free text box. This would not include minor or less specific conditions such as "hypokalemia" or "poor feeding."

For <u>renal diagnoses</u>, please be as detailed as possible:

- Nephrology consult obtained during this admission.
- Acute kidney injury (coded). May also be listed as "acute tubular necrosis (ATN)" or "acute renal failure" Includes ICD-9 codes 584.*
- Urinary tract infections. Please include only if there was a positive urine culture. We are not specifying a particular colony count needed (e.g., >100,000 cfu/ml) or urinalysis criteria (e.g., leukocyte positivity) for the purposes of this study. If there was a clinical diagnosis of a UTI with a corresponding positive urine culture, then code "yes" for UTI.
- Medullary nephrocalcinosis/calcifications/kidney stones. Must be documented on renal ultrasound.
- Congenital abnormalities of the kidney. Please choose all that apply from the list provided. Where applicable, document "right," "left" or bilateral. Use the diagnosis listed in the ultrasound report and/or problem list. If the condition is evaluated on more than one ultrasound during the course of the NICU admission and the graded severity changes, choose the most severe grade (e.g., if the baby has mild left hydronephrosis on first ultrasound but severe left hydronephrosis on follow-up study prior to discharge, list as "unilateral, severe, left").
  - Definitions:
    - Hypoplasia/dysplasia (with or without cysts associated with dysplasia)
    - Multicystic dysplastic kidney (this is a specific diagnosis and is not the same as "polycystic kidney disease" or "cystic dysplasia" or a kidney with just a few cysts. The radiologist or nephrologist will call it multicystic dysplastic or MCDK.)
    - Renal agenesis (congenital solitary kidney)
    - Autosomal recessive polycystic kidney disease (This is a specific diagnosis and not the same as other cystic kidney diseases. This disease usually requires genetic testing for confirmation, but does have a characteristic ultrasound appearance that should be interpreted as "likely ARPKD" by the radiologist or nephrologist. Please code yes if the radiologist states that findings are "consistent with ARPKD" as genetic testing likely will not be available in many cases.)
    - Autosomal dominant polycystic kidney disease (Same as for ARPKD. This is a specific diagnosis and not the same as other cystic kidney diseases. The diagnosis might be made in a baby with cysts on ultrasound who also has a DOCUMENTED history of ADPKD in the family.)
    - Polycystic kidney disease "Unknown" – a baby may be found to have multiple cysts on ultrasound that fall somewhere in between the usual pattern for either ADPKD or ARPKD. If baby has multiple cysts on BOTH sides of the kidney, and the radiologist or nephrologist documents that this is likely PKD but are not sure whether it is AR or

FINAL

AD, then say “yes” to this item. This would NOT include babies with cysts (either one or multiple) in only ONE kidney. Please consult your nephrologist if the information in the chart is not clear.

- Horseshoe kidney
- Renal ectopia (these are kidneys that are found somewhere other than the usual anatomic position, including cross fused ectopia or pelvic kidney)
- Hydronephrosis: If ultrasound lists severity as a range (e.g., “mild to moderate,” choose the most severe recorded
- Ureteropelvic junction obstruction. Must be proven by nuclear medicine scan (MAG-3).
- Hydroureter
- Duplicated collecting system
- Posterior urethral valves
- Vesicoureteral reflux. Must be documented on VCUG or by urology study (cystoscopy, video urodynamics). If more than one VCUG is completed while the baby is in the NICU and the graded severity changes, please use the most severe grade.
- Urethral stricture (not including posterior urethral valves)
- Bladder exstrophy
- Neurogenic bladder
- Prune Belly Syndrome (Eagle-Barrett syndrome)

<u>Renal replacement therapy</u>: *This section should be completed by the nephrologist member of your group.*

Did the patient receive renal replacement therapy during hospitalization? *Yes/no*

- <u>If YES,</u>
  - *How many days did the patient receive any form of renal replacement therapy during the hospitalization? Round to the nearest whole day. Count days for all modalities together (e.g., if a baby was supported with PD for 5 days and CRRT for 6 days then enter 11 days for the total. If the baby was on two modalities in one day, as with transition from one therapy to another) count that as only 1 day in the total.*
  - <u>Modality (please choose all that apply):</u>
    - Peritoneal dialysis
    - intermittent hemodialysis
    - CRRT
    - CRRT with ECMO
    - SLED

If CRRT (including with ECMO), Indicate modality

CVVH

CVVHD

FINAL

CVVHDF
SCUF

If CRRT Indicate machine (please choose all that apply)
Prismaflex
NxStage
Aquadex
Fresenius
In-line filter (for CRRT/ECMO only)

- Type of anticoagulation
    - Heparin
    - Citrate/calcium,
    - None
    - Unknown

FINAL
